# Patient Perspectives on Palliative Care Outreach to Adults Living With Homelessness

**DOI:** 10.1001/jamanetworkopen.2025.52434

**Published:** 2026-01-15

**Authors:** Alexander R. Levesque, Jessica Bytautas, Justine M. Baek, Donna Spaner, Naheed Dosani, Venkata Vanganur, Trevor Morey

**Affiliations:** 1 Dalla Lana School of Public Health, University of Toronto, Toronto, Ontario, Canada; 2 Journey Home Hospice, Toronto, Ontario, Canada; 3 Department of Family & Community Medicine, University of Toronto, Toronto, Ontario, Canada; 4 Department of Family & Community Medicine, St Michael’s Hospital, Unity Health Toronto, Toronto, Ontario, Canada; 5 PEACH (Palliative Education And Care for the Homeless) Program, Inner City Health Associates, Toronto, Ontario, Canada; 6 Mary Heersink School of Global Health and Social Medicine, McMaster University, Toronto, Ontario, Canada; 7 Temmy Latner Centre for Palliative Care, Mount Sinai Hospital, Toronto, Ontario, Canada

## Abstract

**Importance:**

People experiencing homelessness face higher rates of early mortality, multimorbidity, and unmet needs at the end of life, but despite these risks, they often encounter systemic barriers to accessing palliative care. Outreach-based models have emerged to address this gap, yet little is known about how these models are perceived by those receiving care.

**Objective:**

To evaluate a palliative care outreach intervention, the Palliative Education and Care for the Homeless (PEACH) program, from the perspective of patients living with homelessness and life-limiting illness.

**Design, Setting, and Participants:**

This qualitative descriptive study using semistructured interviews with adult PEACH patients was conducted in Toronto, Ontario, Canada, between May 1, 2023, and November 30, 2024. PEACH participants were sampled purposively or conveniently. Interviews were conducted in person or by phone, transcribed verbatim, and analyzed using a reflexive, iterative approach informed by interpretive qualitative description.

**Exposure:**

Receipt of care from the PEACH program during the research period.

**Main Outcomes and Measures:**

Patients’ experiences with the PEACH program, perspectives on program benefits, and evaluation of program strengths and weaknesses.

**Results:**

Fourteen patients between the ages of 20 and 70 years were interviewed: 7 with malignant conditions and 7 with nonmalignant conditions. All but 2 participants were men. The mean (range) duration of contact with PEACH at interview was 18 (3-69) months. Six major themes were identified: life before PEACH, outreach and community care, medical needs, social needs, relational care, and constructive feedback. PEACH addressed barriers by providing low-barrier access and coordination across health care professionals. Participants described improvements in pain and symptom management, mental health, access to supplies, and support with income and housing. The team’s compassionate, person-centered approach, described as relational care, was central to participants’ trust and engagement. Many said they would not be alive without the intervention. Although most feedback was positive, some participants noted areas for improvement, including communication gaps and lack of a single assigned palliative care physician.

**Conclusions and Relevance:**

In this qualitative study of a palliative care outreach intervention from the perspective of people experiencing homelessness, participants emphasized the need for an accessible, compassionate approach that addresses medical and social needs. Findings offer insights for future outreach program development and evaluation.

## Key Points

**Question:**

How do people living with homelessness and life-limiting illness experience a community-based palliative care outreach program?

**Findings:**

Fourteen patients of the Palliative Education and Care for the Homeless (PEACH) program were interviewed for this qualitative study, and 6 major themes were identified: life before PEACH, outreach and community care, medical needs, social needs, relational care, and constructive feedback. PEACH was credited with eliminating systemic barriers, improving physical and mental well-being, and addressing social determinants of health while employing a distinctly compassionate, person-centered approach.

**Meaning:**

Findings of this qualitative study underscore the value of palliative care outreach programs for adults living with homelessness and terminal illness and may inform the development and implementation of future outreach programs.

## Teaser

This qualitative study evaluates the Palliative Education and Care for the Homeless program, a community-based palliative care outreach intervention, from the perspective of adults experiencing homelessness and life-limiting illness.

## Introduction

Internationally, persons experiencing homelessness face substantially higher rates of early mortality.^1,2,3,4^ In Canada, their life expectancy is 30 to 40 years shorter than that of adequately housed individuals.^3,4^ Adults experiencing homelessness also have higher levels of multimorbidity and unique end-of-life symptom burdens.^5,6^ Despite this, they face substantial structural barriers to accessing palliative care.^7,8,9,10^ These barriers include stigma from health care -professionals, inflexible health care systems, insufficient training of health care professionals, and a lack of appropriate care environments.^7,8,9^ In response, the past 2 decades have seen the development of outreach programs for persons experiencing homelessness in several countries.^1,11,12,13,14^ These programs provide psychosocial and medical palliative care in settings where homeless adults live or seek services.^13^

However, the scale of these programs remains limited, operating in only a few regions worldwide.^1,11,12,13,14^ One barrier to replication and scale-up is the difficulty in assessing their direct benefits for individuals experiencing homelessness. Such persons are often considered hard to reach in health research.^15,16^ As a result, research on this subpopulation tends to rely on health care professionals’ perspectives.^1^ Yet in palliative care, patient-reported experiences of well-being are a critical measure. A recent systematic review identified only 1 study, a case report, that assessed a palliative intervention for adults experiencing homelessness based on the experiences of those receiving care.^1,17^

The present study examined the Palliative Education and Care for the Homeless (PEACH) program, a community-based palliative care program for people experiencing homelessness in Toronto, Ontario, Canada, through interviews with PEACH patients. We asked how people living with homelessness and life-limiting illness experience the strengths and weaknesses of a community-based palliative care outreach program. We assessed patient-reported physical, psychological, and social factors associated with PEACH, the barriers participants faced, whether PEACH helped overcome barriers, and potential improvements for palliative care outreach programs. This is the first study, to our knowledge, to assess a palliative care outreach intervention from the perspective of people experiencing homelessness, and the results have implications for future program design and implementation.

## Methods

### Ethics Approval

Institutional ethics approval for this qualitative study was obtained from Unity Health Toronto. This study is reported according to the Consolidated Criteria for Reporting Qualitative Research (COREQ) reporting guideline.^18^ All participants provided written informed consent except in the limited circumstance that a participant was unable to provide written consent without assistance, in which case the consent form was reviewed with the participant and then verbal consent was obtained and documented on the consent form.

### Study Setting

The PEACH program is a mobile, street, and shelter-based interprofessional team of palliative care physicians, psychiatrists, social workers, and nurses who collaborate with health and social service organizations across Toronto, Ontario. Since its founding, PEACH has served over 1000 patients experiencing homelessness. It provides palliative and psychiatric medical care, connects patients with publicly funded home health care supports, helps secure housing, covers health care and medication gaps, and coordinates care with other community and medical supports.^14,19^

PEACH serves unsheltered and vulnerably housed individuals, including those in shelters, government housing, temporary accommodations, and other precarious settings.^20^ Most PEACH patients, and all study participants, meet the Canadian definition of homelessness.^21^ Referral criteria include a life-threatening illness, substantial symptom burden, and difficulty accessing mainstream palliative care.

### Study Design

We conducted an interpretive qualitative description study, well suited to research aiming to inform policy and practice, as it captures participants’ experiences in their own terms.^22,23^ This design is also appropriate for exploratory studies describing a phenomenon rather than generating theory.^22,23^ As the first study, to our knowledge, to examine patient experiences with palliative care outreach, this approach facilitated an inductive analysis and descriptive summary. The research team included qualitative health research experts (J.B.), palliative care specialists (J.M.B, D.S., N.D., and T.M.), and medical trainees (V.V,) with extensive experience working with structurally vulnerable populations in clinical and research contexts.

### Participant Recruitment

Participants were initially recruited through purposive sampling of PEACH patients reflective of the overall PEACH patient population. Recruitment was challenged by medical and social instability, which made interviews difficult to schedule and often cancelled. The team then shifted to convenience sampling, approaching patients when they were stable enough to complete interviews. All patients were approached by PEACH staff, and those interested were later contacted by 1 of 3 members of the study team not involved in patient care (A.R.L., J.M.B., and V.V.), who obtained consent and conducted interviews (eMethods 1 in Supplement 1). Participants were adults over 18 years of age, English-speaking, and able to independently answer interview questions. After their interview, each participant received a $20 coffee shop gift certificate as honoraria, consistent with standards in homeless health research.^24,25,26^

### Data Collection

Data were collected between May 1, 2023, and November 30, 2024, through semistructured one-on-one interviews, conducted in-person when possible or by phone when necessary. To limit the influence of social desirability bias, participants were interviewed by members of the research team not directly involved in patient care (A.R.L, J.M.B., and V.V.). Team members used established strategies aimed at mitigating social desirability bias, such as indirect questioning and probing.^27^ In-person interviews took place in varied settings to accommodate participants, including community housing, shelters, hospital or hospice, and outdoors. All interviews were audio recorded and transcribed verbatim by a member of the research team (A.R.L., J.M.B.). One participant consented for the interview but declined to be quoted directly. Another initially consented but was found to be incapable during the interview, and their data were excluded. Due to the unique circumstances of participants experiencing both homelessness and a life-threatening illness, providing every participant a transcript for review was not logistically feasible. The proposed benefits of member checking are contested, especially when participation is inconsistent.^28^ For this reason, we did not conduct member checking. Interviews followed a guide developed collaboratively and modified iteratively by the full research team (all authors) (eMethods 2 in Supplement 1).

### Data Analysis

Data analysis occurred alongside data collection. All transcripts were imported into NVivo, version 14 (Lumivero). Following a reflexive, iterative process informed by interpretative qualitative description, coding was guided by and adapted to the data.^22,23^ Five members of the research team (A.R.L., J.B., D.S., N.D., and T.M.) began by individually coding 4 transcripts using an inductive approach,^22,23^ and then met to compare and refine initial codes. Four additional transcripts were coded using the revised codes. Through this deliberative process, refined and emergent codes were grouped into subthemes and then major themes. The Figure illustrates this process for the major theme of relational care. All transcripts were recoded using this framework. Interpretive sufficiency was achieved through ongoing team discussions and iterative engagement with data. The research team (all authors) determined that additional interviews were unlikely to yield new insights or deepen interpretation. Final themes were collaboratively refined and agreed on.

**Figure.**
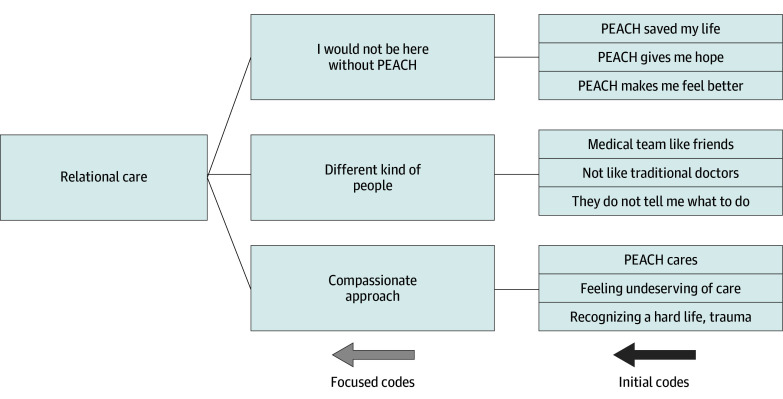
Emergence of the Major Theme, Relational Care PEACH indicates Palliative Education and Care for the Homeless.

## Results

Table 1 presents demographic characteristics of the 14 participants. Ages ranged from 20 to 70 years. All but 2 participants were men, which is reflective of the PEACH patient population.^20^ Malignant and nonmalignant terminal conditions were equally represented (ie, 7 patients in each). Most participants lived in a shelter or in community or social housing at referral and were referred to PEACH by physicians. Duration of contact with PEACH at the time of interview ranged from 3 to 69 months (mean, 18 months).

**Table 1.** Demographic Characteristics of Interview Participants

Participant No.	Gender	Age decade	Palliative diagnosis	Housing status at time of referral to PEACH	Source of referral to PEACH	Duration of PEACH contact at time of interview, mo
1	Man	60s	Lung cancer	Shelter	Shelter physician	6
2	Man	60s	Prostate cancer	Shelter	Shelter physician	7
3	Man	70s	Prostate cancer	Shelter	Palliative care physician	4
4	Man	50s	Liver cirrhosis	Shelter	Shelter physician	7
5	Man	20s	Complications of HIV	Rooming house, group home, or transitional housing	Palliative care physician	17
6	Man	60s	COPD	Community or social housing	Social worker	69
7	Man	60s	Lung cancer	Community or social housing	Palliative care physician	9
8	Man	50s	COPD	Market rent apartment	Patient’s family member	14
9	Woman	60s	COPD	Community or social housing	Inpatient hospital physician	24
10	Man	60s	Lung cancer	Market rent apartment	Social worker	5
11	Man	50s	Colon cancer	Community or social housing	Shelter physician	55
12	Man	40s	Liver cirrhosis	Market rent apartment	Family physician	28
13	Man	60s	Lung cancer	Rooming house, group home or transitional housing	Palliative care physician	3
14	Woman	40s	Complications of HIV	Community or social housing	Oncology team member	6

Abbreviations: COPD, chronic obstructive pulmonary disease; PEACH, Palliative Education and Care for the Homeless.

Six major themes were identified: life before PEACH, outreach and community care, medical needs, social needs, relational care, and constructive feedback. Table 2, Table 3, and Table 4 provide selected illustrative quotes; additional supplementary quotes are presented in eTables 1-3 in Supplement 1.

**Table 2.** Participant Quotes on Life Before PEACH

Subtheme/Participant No.	Quote
Hard life	
1	To give you a li’l bit of background, my wife got ALS [amyotrophic lateral sclerosis] about 5 years ago, and that’s it. Died. And after that I ended up homeless.
1	*If you didn’t have access to PEACH, what do you think or how would you think you would’ve addressed your needs?* Oh I would’ve given up. I would’ve given up after the wife passed. I-I- I- was really, really in a bad place, and having a cancer diagnosis is horrible. I would’ve given up, for a 100%, I would’ve given up for sure. No question about it, I wouldn’t made it through.
2	[My daughter] ended up dying and uh my ex didn’t even phone me about the funeral or something. My son found out on social media and stuff, he lives in Vancouver, he phoned me… and he said, “I phoned you, Dad, because [she] died of an overdose and the funeral’s tomorrow, and I know [Mom] probably didn’t want to tell you.” So I went to the funeral, and I think she was surprised I was there.
3	When I was inside, I was only going to see the cancer clinic pretty much every week; and the doctor there said, “I will make sure that I look after you, and that I am going to make you better,” right? He said that I promise that. But, when I went back to the institution, everything that he had prescribed and done for me, they wouldn’t allow it.
6	*And do you know why you’ve been receiving care from PEACH or why you were referred initially?* Because at that time my social worker knew I wasn’t going to make it on my own. I was barely making it, so… I was in pretty rough shape… So, you know, they’ve been there ever since.
7	I have this disease, nontuberculosis mycobacterium avion… Anyway the MAC [mycobacterium avium complex] wouldn’t go away. There’s a 3-antibiotic treatment for MAC, and after 4 years of being on this treatment for it, it was [apparent] it was never going to go away. I was frequently getting infections and had pneumonia, and sometimes the antibiotics would work and sometimes they wouldn’t. And I said to my lung specialist, there has to be something to this. Something is happening in there that’s making this all not work. So she sent me for massive blood tests… [and] they found out I have multiple myeloma.
8	My life is completely changed. I literally lost more than half my body weight. I had to learn how to walk again and learn how to eat again. In February, I was just unloading trucks, and now I’m walking with a walker.
13	I was hit by a car in 2013, and I have a broken neck as well. I’ve got some bolts, you know. They bolted my neck to my spine, and the pain from that is just outrageous. I’m in constant pain all day every day, and I was getting such a low amount of pain help that it just wasn’t working.
Substance use	
4	I continued drinking really hard, and I continue drinking really hard, like, I’m at three 26s a day and then some… I was just trying to get out of my mind, just to get away, and all I do is really hurt myself. Like my liver’s gone... [a] whole lotta stuff in my stomach, like’s, all screwed right up.
9	I’m a recovering addict. People are knocking on my door wanting to get high with me. It’s really hard. I only have 3 months clean.
9	I was sober for 7 years. I went to rehab, and I was sober for 7 years. I relapsed 5 years ago, but I was sober for 7 years.
9	Like, there was 8 kids in my family, and me and my brother were the only ones my dad beat, and we both became addicts. The others didn’t even take a drink in their life or a cigarette. That’s not a coincidence. That’s our trauma.
Previously encountered barriers	
3	You’d have hours where you would have to wait… I always had to run up to the hospital to see a doctor when I’m having problems and then sit there… sometimes for 4 or 5 hours. Then you go in, and then it’s another hour or so and just for a problem.
3	I wouldn’t be where I am right now, not a chance. [PEACH] are the ones that took the time to—nobody else thought about the power of getting my meds. The pharmacy didn’t think about it. The [other] doctors looked at it, but they didn’t think about it.
8	I would still be living at my old place. Probably locked in my apartment, no way to breathe because I would not be going anywhere. I know… I know myself. Yeah, I wouldn’t be going anywhere. [If it wasn’t] for PEACH team coming here or coming to me and helping me, I would not be seeking help.
8	I’ve worked with 4000 other people, and basically you’re told to fuck off all day long. Sorry for the language but that’s exactly what happens when I’m out in the world. But [PEACH], they come to my place, and I don’t know, they’re just so, so good. And I’m not used to that. Anytime I’ve walked into a walk-in clinic or anything like that, they’re just so, so impersonal. And with these people, it’s just like they’re caring!
12	I will be down, because some of the time, I can’t buy my medication, as I have to buy my medication myself, and I’m not working. So sometimes, as I said, I get various aches, and somebody might sponsor me.
14	Oh yeah, they [involuntarily admitted] me for 4 months in hospital. That’s how connected with psychiatry I was… I’ve never had a psychiatrist speak with me before… In fact, they wouldn’t even look at me. I’ve never had one speak to me… I’ve been formed a bunch of times [involuntarily detained in hospital, either for psychiatric assessment or psychiatric treatment], and I’ve never had anyone speak to me. They just look at me and walk away. Then I can’t go anywhere, and I can’t smoke, and I can’t, you know what I mean, I can’t get any food, and I can’t get anything.
14	I have a lot of doctors, so someone’s always catching something. But like I said, they’re all disconnected. So the PEACH team just sort of steps in and says, yeah, we can just [do it]. They don’t say, “That’s not my area,” whereas all of my other doctors are kind of on their own focus.
14	I have asked every fucking doctor I’ve ever had since they’ve been locking me up… and no one has ever even taken a look [at the disability form], but then they never do… I’ve asked every doctor. I’ve printed it off and handed it to people. Nobody’s ever helped me out.
14	Yeah, it’s hard to talk to doctors. They have a certain idea in their head about your care, and it’s sort of hard to bring up your own, you know what I mean?
Stigma	
1	I kind of kept it to myself… It’s hard to tell somebody, “Hi. I have lung cancer. Can I go to your place and die?”
3	I came out really worried about seeing people, thinking they know you’re out of prison.
13	*Do you think people in the health care system, like doctors or nurses, have ever treated you differently because you use?* Well, let’s just… you’re frowned on, you know what I mean? Pretty much everywhere… because they look at [you] like if you weren’t doing this, you wouldn’t be [in this situation].
14	*How were you taking care of your mental health before the PEACH psychiatrist? Were there people helping you with that at all?* I was afraid to touch it at all because I was afraid of getting locked up in a hospital again if I opened my mouth. So I was afraid to say anything to anybody.

Abbreviation: PEACH, Palliative Education and Care for the Homeless.

**Table 3.** Participant Quotes on PEACH Patient Services

Theme/Subtheme	Participant No.	Quote
**Theme: Outreach and community care**		
Connecting with PEACH	7	Initially it started with me trying to find out about applying for MAID [Medical Assistance in Dying], and I was directed to initially speak with [the home care coordinator]… Anyways, that’s how it all got started.
	8	Actually, my sister-in-law got [me] connected. She knew somebody, a nurse from the PEACH team.
	11	Actually, they visit[ed] me, at the hospital [where] I [got] the surgery.
	13	They showed up right at the hospital. Yeah, it was a nice feeling. I was able to relax a bit. That’s a big help when it comes to pain, you can relax a bit and it takes some of that pain away. *How soon after you went home did the PEACH team come and see you?* The next day.
Ease of access	1	Every time I have the slightest problem, I call [the health navigator] right away. And she really fixes it or points me in the right direction.
	3	I got all the numbers. I just make a call to PEACH. Yes, they’re so friendly, yes. “Who do you need to see? Right?” And I say “Okay, can I see this person, can I get some more of this,” or “Can I change that,” or “I don’t feel well.” “Okay we’ll send the doctor in or whatever.” Or I need more of the pamper things there… they’ll go to work on that. Anything I need, I call and they’re right there.
	12	Sometimes when I’m sick, they’re the only one I can call, and they will be there for me in a jiff.
Home care	1	I’m in a [shelter] program. I don’t, like, really get around. It’s a big place. There is a lot of people here. But [PEACH], they go through all the red tape and come right to my room.
	3	One of the [PEACH] workers comes in and does my vitals and everything all the time, and we talk and see if there’s any problems. And so she comes every week, and my doctor’s right there for me for anything I need.
	14	I’m really... all over the place. And it was good for me to not have to worry about getting out of the house, about getting money, and about being presentable in public.
Care coordination	1	*Can you describe a little bit about what the PEACH team does for you?* Everything that is cancer related. From setting up the appointment to dealing with the [oncologists] to getting the transportation, making sure people are coming with me, that I’m well supported. Everything. And excellently!
	7	I give kudos to the PEACH team for how quick they reacted to the initial conversation with sending over doctors, inquiring what kind of care I needed for PSWs, and things like that.
	12	They offer transportation to wherever I want to go. Sometimes it’s just to the hospital, so I tell them I can just jump on the bus… But they always insist to come and help.
Collaboration	7	One of the [PEACH] doctors that came to see me was familiar with my oncologist and had communications with her before, and that helped.
	9	I wanted to go home, and I wasn’t ready. I couldn’t function at home. So finally PEACH found this place for me, this hospice, and I’m so happy… And then 3 of the doctors here [at the hospice] had been to my place with PEACH.
	13	Yeah, I have a counselor that looks out for me and they converse a lot [with the PEACH team]. Yeah, it’s great because they have this connection, and the worker I have here is also… pretty good, too, and she comes to me with any info. Anything I have, she’s right here.
**Theme: Medical needs**		
Pain and symptom management	3	When I was having the problem with my bowels and things like that, of course I brought it up to the other doctors, but they didn’t really have much to say about it. “Well that’s interesting.” Yeah, that’s interesting all right (laughing). Yeah, I poop in the bed. Nope the doctor here, he got me fixed up there so that hasn’t happened now in close to a couple months now.
	4	*And if you were not served by PEACH right now, what do you think your health would be like?* I think I’d be really hurting. For sure, I’d be really hurting.
	13	If it wasn’t for them, I’d be nowhere near where I am… Well, because I was lying in the bed crying all day long because of the pain, and now, I can actually sit here and talk to you. Three to 4 months ago, I wouldn’t have been able to do that.
Primary care	1	Last week I had a bad reaction to the chemo treatment, and I had… my left leg started getting bigger and bigger and bigger and bigger, and like, right away, they adjusted all the meds.
	8	The PEACH team got me... on a nutrition diet, I guess. Make sure that I’m putting enough calories in and stuff. So it’s good. They, like, make sure I get, like, Boost and stuff delivered to the house.
	14	I had a bladder infection I couldn’t get rid of. They started writing prescriptions for that because I just wasn’t getting to a bladder doctor.
Medical supplies	7	They helped me with a walker, a wheelchair—put me in contact with the provincial program that got me my scooter. So, yeah, as far as equipment that I could use to give me more mobility, because of the lung issues, yeah, they were very, very helpful.
	9	They got me a hospital bed, they got me a walker. If I don’t have the money, they’ll get it. They even got me a new set of sheets for my hospital bed.
	9	They send me diapers, and they send me my colostomy supplies at home. What was happening with the first nurse I had is I went 11 days without colostomy supplies, and I couldn’t eat. And then the next time was 6 days. The new nurse I had [through PEACH], I get them before I need them.
Mental health care	1	There’s also the subsequent [psychological] fallout from the moment that you know you’re going to die, right? The mental, the everything. [PEACH] have taken care of everything.
	8	They got me hooked up with a [psychiatrist], which is okay. I never thought I would have to deal with [this], but it was something I really need and I’m actually surviving because of it. And without PEACH, I can honestly say, I wouldn’t be talking to this person.
	14	Well, my mental health would not be as good [without PEACH] because the psychiatrist has been really, really great… I’d just be a lot more frantic and a lot more… It’s good grounding to get a lot of stuff off my chest I can’t say to other people.
Harm reduction	9	I don’t feel like they’re working for me, you know. They’re giving me a service. I think they’re my friends. And my nurses, you know, I can talk to them about my addiction, even if I used an hour before, I would still tell them.
	13	*How would you have addressed your needs if you didn’t have the PEACH team? For pain and things like that?* I would’ve went to the street. You can only take so much pain, it just, it wins. All you can do at the most is maybe calm it for an hour or so, you know what I mean. And I haven’t had to as of yet, and with the PEACH guys, I don’t think I’ll ever have to… Because, considering where we are, to go to the street is so easy, you know. It’s a given. And I can still say no to all that stupidness.
**Theme: Social needs**		
Financial support	1	[The PEACH health navigator] is outstanding. Like going through, I had real hard time with [the Ontario Disability Support Program]. She just kept knocking at it till I got in. I got in like about a month ago.
	12	I will be down [without the PEACH team], because some of the time I can’t buy my medication, as I have to buy my medication myself, and I’m not working. So sometimes, as I said, I get various aches, and somebody might sponsor me. Or if I can’t get dispensed, I’ll call [the PEACH health navigator] and she would, she would make sure that I get it.
	14	[PEACH] came and helped me fill out the disability tax credit, which was good… I asked every [previous] doctor to [complete] it and everyone said they were going to do it, and no one ever did it.
Housing	2	Just to get in this place and get a place, a lot of people wait years. I mean I’ve been in the shelter system for almost 3 years. But still, people [wait] 7, 10 years.
	8	They’re currently helping me right now with my house. Cause right now I moved with my brother and sister-in-law. Cause they live in a house and there’s no stairs. They put me on a ground level of the house, which is nice. I have no stairs or anything like that, and right now, as we speak, I’ve actually gotten 2 applications in for 2 apartments. And yeah, that’s through PEACH. PEACH has helped me out greatly on that. They got me through Toronto housing ahead of time and beautiful housing.
	9	PEACH found this place for me, this hospice, and I’m so happy. Like my apartment was dangerous for me. I’m a recovering addict. People are knocking on my door wanting to get high with me. It’s really hard; I only have 3 months clean.
Food security	10	[The PEACH doctor], she was the one coming there, sending food—how do you call it? The chocolate stuff. And it was a bit cold that time, she bring me sleeping bags.
	12	The gift cards, they help me a lot when [they] send them to me, bring them to me, and I can go and shop… I know that I have something to eat, something to drink. I can give thanks and say the PEACH team is here for me because I’m not going to my bed hungry. I’m not gonna say that that I’m hungry when they give me [a] gift card to go and shop.
Connecting with loved ones	3	PEACH ended up sending me and my family over to [a] restaurant. It was so nice, and they paid for the meal for all of us and the ride to get there and back. That was awesome. That was the last night before my family left, so it was a good ending to a 3 day stay.
	9	PEACH gave me hope. Additionally… my grandchildren are going to remember me you know. He’s only 2 months old, but he’s going to remember me.
**Theme: Relational care**		
Compassionate approach	3	They came in and took charge of me and took care of me like I was, you know, their only patient in a way. That’s how they made me feel.
	4	They really kind, they’re generous, and they know what’s wrong with you before you do.
	7	Like I said, the compassion and care that the doctors and nurses had, and really I believe wanted to help, do the best that’s in their abilities to help me do the best I can to get through and have the best quality of life going toward [the] end of it. So that’s the best thing.
	8	It almost feels like I’m privileged, you know what I mean? Like I don’t know if I am, but it almost feels like I’m getting, you know, privileged, taken care of more. I hope everybody is getting taken care of, and I’m sure they are. It’s just they’re so [caring].
	9	That’s what PEACH gave me. Hope. PEACH gave me hope.
	12	The PEACH team… I feel, I feel love, I feel trust, and I can, I can say anything. I can ask them any question, and I can say anything, and they will give me an answer, and they’re always there. So the PEACH team is a blessing.
Different kind of people	4	And, like, you know, they don’t bother you. They just help you, like, they don’t bother you saying, “You have to do this, and you have to do that, you have to hear me, and you have to do this.” They don’t do that, you know? They just let you do what you got to do and… they cheer for you.
	6	Nobody else is there for you when you need them no matter what, you know.
	7	The best thing that I found about the PEACH team is the compassion that they had. I believe that everybody that I dealt with truly had the ambition to help. And that was the best thing, was that they wanted to help. And you don’t find that everywhere. In hospitals, it’s the choice of doctors and nurses… Best thing, knowing that somebody gives a shit. That’s great.
	8	Yeah, they are amazingly caring. I’m not used to this. I’ve worked with 4000 other people, and basically, you’re told to fuck off all day long. Sorry for the language, but that’s exactly what happens when I’m out in the world. But these people, they come to my place, and I don’t know, they’re just so, so good. And I’m not used to that. Anytime I’ve walked into a walk-in clinic or anything like that, they’re just so, so impersonal. And with these people, it’s just like they’re caring!
	14	They [have] like a less stern outlook on life… They just [make] things easier, you know… More approachable.
Wouldn’t be here without PEACH	1	Truthfully, I wouldn’t be around if it wasn’t for PEACH… I’d be dead. I would’ve given up. It’s too complicated. Oh yeah, for sure. There’s not even a question in my mind.
	2	I could be dead. Who knows, huh? They’ve done so much for me and everything. It was like I had someone on my side.
	3	I wouldn’t be where I am right now, not a chance. They’re the ones that took the time to—nobody else thought about the power of getting my meds… It was the doctor at PEACH who worked it out… I wouldn’t be sitting up like this going and exercising. I’d still be laying on my bed sleeping most of the day and night, yeah.
	7	I don’t know where I’d be without them. It got to the point where I could not function alone in my own apartment.
	9	I wouldn’t be here today if it wasn’t for PEACH. I’d be dead.

Abbreviations: PEACH, Palliative Education and Care for the Homeless; PSW, personal support worker.

**Table 4.** Participant Quotes on Constructive Feedback

Subtheme/Participant No.	Quote
Gratitude	
1	*Is there any area that you feel like that they could improve or anything that you think that they could work on as a team together?* No, I really don’t, I really don’t. Every aspect so far has been outstanding. Like, really. I’m not just saying that.
2	I’m glad I had a chance to talk about PEACH, how great they are. I’m really, really happy with them.
4	Yeah, they’re helping me with everything. Yes, they’re helping me with everything. Name it, they help me with it. They’re just unbelievable. To me, they are, to me, they’re just great.
8	And you know I’ve never been a doctor person, and they’re making it easy for now, which I appreciate.
9	I’m so grateful to PEACH when I tell you I’m happy. You can tell. When I talk about PEACH, I’m like… I love them. I love them.
12	They’re doing a wonderful job… I wouldn’t change anything. Just give them [my] blessing, and [I’m] grateful for them, thankful for them.
13	Just a really super huge hug and thank you. I mean that too, I really do mean that. I haven’t been so serious in my life until this point and just that, you know, for the extra couple of years I may get.
PEACH would be a good service for others	
1	[PEACH would be good for] even the people that are housed that don’t have like a very good support system. Sure, you should expand it.
9	*Any other kinds of people you feel PEACH would be good for?* Well homeless people, homeless people. People who are struggling to come off of a drug. Especially for women. Men have many, many resources out there. Not women, not women.
14	I have one friend who was just in hospital, and she had pneumonia… And I just think, why can’t these great people who sort of come to the building… and I could just point her out and say, “She needs to be talked to.” I think they could’ve made a huge difference, and she wouldn’t be dead right now.
Areas for improvement	
7	There was no appointment set, and I didn’t like that part of it at all. I’m an organized guy, I want appointments set, and that’s the way I operate anyways. And it wasn’t working that way with the [PEACH] program.
7	I mean to have a regular doctor look after your needs rather than different ones all of the time would be great.
9	The only thing I find hard is that every time I have different doctors, and you feel like you have to tell your story again. You get kind of tired [of] telling your story.
13	I don’t think anyone around here has heard of the PEACH team. I almost think they’re brand new.

Abbreviation: PEACH, Palliative Education and Care for the Homeless.

### Life Before PEACH

Although participants were not asked explicitly about their life before PEACH, nearly all volunteered this information. Within this broader theme, 4 subthemes emerged: hard life, substance use, previously encountered barriers, and stigma (Table 2).

All participants described severe hardships before PEACH, including homelessness, incarceration, serious illness, bereavement, and trauma. For example, 1 participant stated “My wife got ALS [amyotrophic lateral sclerosis] about 5 years ago, and that’s it. Died. And after that I ended up homeless.” Many also endured long waits for housing, unsafe conditions, and limited social support.

Several participants had a history of substance use, often linked to trauma, health struggles, and marginalization. As 1 participant described, “There was 8 kids in my family, and me and my brother were the only ones my dad beat, and we both became addicts... That’s not a coincidence. That’s our trauma.”

Before PEACH, most participants encountered systemic health care barriers, including long waits, limited access to medications, and dismissive treatment. Some also felt stigmatized due to substance use, past incarceration, or housing status. One participant shared, “You’re frowned on, you know what I mean? Pretty much everywhere… because they look at [you] like, if you weren’t doing this, you wouldn’t be [in this situation].”

### PEACH Patient Services

The themes outreach and community care, medical needs, social needs, and relational care reflect different aspects of the services provided by PEACH (Table 3).

#### Outreach and Community Care

Analysis revealed 5 subthemes within the theme of outreach and community care: connecting with PEACH, ease of access, home care, care coordination, and collaboration. Participants highlighted a low-barrier referral process, often initiated by allied health professionals or case workers. The PEACH team frequently connected with participants and their inpatient care teams, which allowed for care to be transitioned immediately after discharge from the hospital. PEACH remained accessible, offering a direct line of communication for nearly any issue. As a participant described, “Every time I have the slightest problem, I call [the PEACH Health Navigator] right away. And she really fixes it or points me in the right direction.”

Participants also emphasized the value of receiving medical care in their community setting, addressing challenges related to mobility, disability, finances, and navigating health care spaces. One participant explained, “I’m in a [shelter] program. I don’t, like, really get around. It’s a big place. There is a lot of people here. But [PEACH], they go through all the red tape and come right to my room.”

Many also relied on PEACH to coordinate their care, managing appointments, arranging transportation, and collaborating with other health and social service organizations. As 1 participant said, “[PEACH does] everything that is cancer related. From setting up the appointment to dealing with the [oncologists], to getting the transportation, making sure people are coming with me, that I’m well supported, everything. And excellently!”

#### Medical Needs

Participants spoke to 5 subthemes within the theme of medical needs: pain and symptom management, primary care, medical supplies, mental health care, and harm reduction. As a palliative care team, pain and symptom management is central to PEACH’s work. Many participants felt PEACH addressed severe symptoms that had gone untreated due to systemic barriers and stigma. One participant shared, “If it wasn’t for them, I’d be nowhere near where I am… Well, because I was lying in the bed crying all day long because of the pain, and now, I can actually sit here and talk to you. Three to 4 months ago, I wouldn’t have been able to do that.” Beyond symptom management, participants described PEACH meeting other medical needs, often stepping into primary care roles and providing essential medical supplies to help manage serious medical conditions comfortably.

Several participants also emphasized the importance of embedded mental health care, which helped them overcome longstanding barriers to psychiatric support. As 1 participant shared, “There’s also the subsequent [psychological] fallout from the moment that you know you’re going to die, right? The mental, the everything. [PEACH] have taken care of everything.”

Finally, a few participants spoke indirectly to harm reduction, a core principle of PEACH’s care. Some credited PEACH with helping them use substances more safely or reduce substance use.

#### Social Needs

Participants frequently spoke to PEACH’s role in addressing their social needs. Four subthemes emerged: financial support, housing, food security, and connecting with loved ones.

PEACH addressed financial challenges by helping participants access disability benefits, medication coverage, and providing essential items. Many participants had struggled to find such support in the broader health care system: “[PEACH] came and helped me fill out the disability tax credit, which was good… I asked every [previous] doctor to [complete] it, and everyone said they were going to do it, and no one ever did it.”

Participants also emphasized PEACH’s role in helping them find safer, more stable housing, which often improved their overall well-being. One participant shared, “They’re currently helping me right now with my house… and right now as we speak, I’ve actually gotten 2 applications in for 2 apartments. And yeah, that’s through PEACH. PEACH has helped me out greatly on that. They got me through Toronto housing ahead of time and beautiful housing.”

While financial support and housing were most frequently mentioned, other participants received help accessing food or nutritional supplements. PEACH also supported participants in reconnecting with loved ones: “PEACH gave me hope. Additionally, my grandchildren are going to remember me you know. He’s only 2 months old, but he’s going to remember me.”

#### Relational Care

All participants commented on PEACH’s overall approach to care, which we refer to here as relational care. Three subthemes emerged within this overarching theme: compassionate approach, different kind of people, and wouldn’t be here without PEACH.

Participants described PEACH staff as more than just health care professionals; they were trusted individuals who made participants feel valued and supported:

*The best thing that I found about the PEACH team is the compassion that they had. I believe that everybody that I dealt with truly had the ambition to help. And that was the best thing, was that they wanted to help. And you don’t find that everywhere. In hospitals, it’s the choice of doctors and nurses… Best thing, knowing that somebody gives a shit. That’s great.*

Most participants felt this compassionate approach stood in stark contrast to their past experiences in the health care system. PEACH staff were seen as warm, approachable, and genuinely committed to patient-centered care:

*Yeah, they are amazingly caring. I’m not used to this. I’ve worked with 4000 other people, and basically, you’re told to fuck off all day long… But these people, they come to my place, and I don’t know, they’re just so, so good. And I’m not used to that. Anytime I’ve walked into a walk-in clinic or anything like that, they’re just so, so impersonal. And with these people, it’s just like they’re caring!*

Several participants also credited PEACH with saving their lives. As 1 participant stated, “I wouldn’t be here today if it wasn’t for PEACH. I’d be dead.”

### Constructive Feedback

Three subthemes emerged within the broader theme of constructive feedback: gratitude, PEACH would be a good service for others, and areas for improvement (Table 4). Participants were explicitly asked to offer critical feedback to help improve the program. Despite this, most shared only commendations. Many responded to questions about limitations with expressions of gratitude, such as “They’re doing a wonderful job… I wouldn’t change anything. Just give them [my] blessing, and [I’m] grateful for them, thankful for them.” Several also expressed a desire for more people in similar situations to have access to PEACH.

PEACH’s team-based model meant participants were sometimes engaged by multiple health care professionals, and a couple of participants expressed a desire to have a single, consistent doctor: “I mean to have a regular doctor look after your needs rather than different ones all of the time would be great.” Another concern included a lack of public awareness of PEACH in various settings across the homelessness sector.

## Discussion

This qualitative study explored patient perspectives on a palliative care outreach intervention for adults experiencing homelessness. Participants described many positive factors across 4 domains: outreach and community care, medical needs, social needs, and relational care. Feedback was overwhelmingly positive, with many participants crediting the intervention with improving, and in some cases, saving their lives.

Only 1 prior study, a case report, has examined a palliative care intervention for people experiencing homelessness from the perspective of the participants.^17^ Ours is the first study, to our knowledge, to evaluate patient perspectives on a palliative care outreach team. We had to overcome substantial challenges in conducting interviews in homes, shelters, and on the street. People experiencing homelessness face substantial barriers to traditional palliative care,^7,29,30^ and participants emphasized PEACH’s accessibility, communication, and care coordination. While previous research suggests persons experiencing homelessness emphasize symptom management needs at the end of life,^7,30^ our findings also underscored unmet primary care, medical supply, and psychiatric needs. One review identified only 1 palliative care intervention that directly addressed social needs for this population, primarily through hospice admission and family reconnection.^1,31^ In contrast, participants in our study emphasized the value of income supplementation and food security, alongside stable housing and social connection, when facing advanced illness. Participants also spoke extensively about the PEACH team’s compassionate, empathetic, trauma-informed approach and accessible demeanor. This emphasis on compassion aligns with previous qualitative research on end-of-life perspectives among people experiencing homelessness.^30,32^ In addition to considering accessibility, medical, and social needs, future palliative care outreach interventions should adopt care philosophies that are responsive to the unique needs of persons experiencing homelessness.

The results of our study offer foundational insight for developing palliative care outreach teams for equity-deserving populations in other jurisdictions. Further research evaluating such programs would strengthen the evidence base and support broader policy and funding decisions. Additional studies are needed to assess the effects of palliative care outreach on measurable health outcomes, such as symptom scores, functional performance, and hospitalization rates. Finally, as this is only the second study that we are aware of to examine end-of-life care interventions from the perspective of persons experiencing homelessness, this work underscores the need for continued research in this area.^1^

### Limitations

This study has limitations. There was an overrepresentation of men among participants; only 2 of 14 respondents were women. While this number is reflective of the overall PEACH patient population, women comprise nearly 50% of Canada’s homeless population.^33^ Our recruitment aimed to obtain a sample reflective of the PEACH patient population, but future studies could consider alternative approaches to increase the representation of women. Results may also be influenced by the research team’s combination of purposive and convenience sampling. However, this approach was necessary given the unique challenges in reaching this population, including severe illness, housing instability, substance use, and mental health issues. Although the study aimed to elicit critical feedback, responses were overwhelmingly positive, limiting conclusions about weaknesses of the intervention and opportunities for improvement. Participants may have been concerned criticism would jeopardize their care, despite the mitigatory practices employed by the research team. Nonetheless, critical feedback gathered aligns with key themes in our findings and previous literature, including the importance of consistent communication, social support, and trusting relationships.

## Conclusions

In this qualitative study of a palliative care outreach intervention from the perspective of adults experiencing homelessness, participants emphasized key aspects of the intervention, including compassionate, accessible care, and attention to unmet physical, psychological, and social needs. These findings can inform future outreach-based palliative care programs for these vulnerable members of society.

## Notes

Supplement 1. **eMethods 1.** Participant Consent Form **eMethods 2.** Interview Guide **eTable 1.** Supplementary Quotes – Life Before PEACH **eTable 2.** Supplementary Quotes – PEACH Patient Services **eTable 3.** Supplementary Quotes – Constructive Feedback

Supplement 2.
Data Sharing Statement
