# Supplementary material for: Patient Perspectives on Palliative Care Outreach to Adults Living With Homelessness
Source: JAMA Netw Open. 2026 Jan 15;9(1):e2552434. doi: 10.1001/jamanetworkopen.2025.52434 (PMC12809365; doi:10.1001/jamanetworkopen.2025.52434)
Supplement: Supplement 1. — eMethods 1. Participant Consent Form eMethods 2. Interview Guide eTable 1. Supplementary Quotes – Life Before PEACH eTable 2. Supplementary Quotes – PEACH Patient Services eTable 3. Supplementary Quotes – Constructive Feedback [file jamanetwopen-e2552434-s001.pdf]

Supplementary Online Content

Levesque AR, Bytautas J, Baek JM, et al. Patient perspectives on palliative care outreach to adults living with homelessness. *JAMA Netw Open*. 2026;9(1):e2552434.
doi:10.1001/jamanetworkopen.2025.52434

eMethods 1. Participant Consent Form

eMethods 2. Interview Guide

eTable 1. Supplementary Quotes – Life Before PEACH

eTable 2. Supplementary Quotes – PEACH Patient Services

eTable 3. Supplementary Quotes – Constructive Feedback

This supplementary material has been provided by the authors to give readers additional information about their work.

© 2026 Levesque AR et al. *JAMA Network Open*.

eMethods 1. Participant Consent Form

CONSENT TO PARTICIPATE IN A RESEARCH STUDY

TITLE OF PROTOCOL: Experiences of Structurally Vulnerable Patients receiving Palliative Care

INTRODUCTION:
You are invited to consider participating in a research study that aims to explore your experiences with palliative care and the Palliative Education and Care for the Homeless Team. Before agreeing to take part in this research study, it is important that you read the information in this research consent form. It includes details we think you need to know in order to decide if you wish to take part in the study. If you would like this form to be read to you a member of the research staff can read it to you. If you have any questions, please ask the study staff. You should not sign this form until you are sure you understand the information. All research is voluntary.

PRINCIPAL INVESTIGATOR:
Trevor Morey, MD, CCFP(PC)
Palliative Care Physician, Inner City Health Associates

CO-INVESTIGATORS:
Justine Baek, MD, CCFP, Palliative Care Fellow
Contact: 647-967-3735 (Monday to Friday 9:00 a.m. - 5:00 p.m)

Lilian Robinson MD, CCFP, Palliative Care Fellow

Jessica Bytautas, PhD Candidate

Alexander Levesque, MD

Venkata Vanganur, MSc Candidate

Donna Spaner, MD, CCFP, FCFP, MScCH

Naheed Dosani, MD, CCFP (PC)

CONFLICTS OF INTEREST:

There is one potential conflict of interest: The Principal Investigator, Dr. Trevor Morey, and co-investigators, Dr. Donna Spaner and Dr. Naheed Dosani, are affiliated with the Palliative Education and Care for the Homeless (PEACH) program. However, this will be managed as the interviews and data analysis will be conducted by researchers who are not affiliated with the PEACH program. Agreeing or declining to participate will not impact the quality of care you receive from the PEACH team. PEACH healthcare providers will not have any access to the interviews, transcriptions of interviews or any identifiable data during the study period.

STUDY SPONSOR:
This study is not sponsored.

PURPOSE OF THE RESEARCH:
We are hoping to understand the health care needs of clients receiving care at PEACH and the barriers they face in accessing health care. Our goal is to find ways to improve care for clients receiving care at PEACH in the future.

DESCRIPTION OF THE RESEARCH:
We are inviting clients who are receiving care from PEACH to sit down for a 30-60 minute interview to explore your experiences with PEACH. The goal of this project is to find out what is done well and what can be improved at PEACH and other similar care teams. The interview will be arranged in person, via telephone or via video based on your preference with one of the researchers at a time that works best for you. No one who provides care at PEACH

© 2026 Levesque AR et al. *JAMA Network Open*.

will be conducting the interviews or have access to what is said in the interviews. This is done so that your care is not affected by participating in our research study.

We may also be collecting information about clients that agree to be interviewed, however, the information will not have your name. This will include information such as your age, gender, ethnicity, number of years of education, diagnosis, previous place of residence, type of provider who referred you to the PEACH.

POTENTIAL HARMS: It is possible that some questions might be uncomfortable to answer, or might make you feel strong emotions. This interview is completely optional. If you decide to participate, you can change your mind at any time and stop the interview without any negative consequences. If you choose to remove yourself and your interview from the study, all information you have provided during the interview will be withdrawn from the study and destroyed. If you are uncomfortable during this interview, we will take steps to inform your care team urgently in order to increase supports for you as a client. Please be assured that the information provided by you will be kept strictly confidential by the team unless we believe you are at risk of harm to yourself or to others. If there is reason to believe you are at risk of harming yourself or at risk of harming others, we are required to report this information to appropriate medical care providers.

POTENTIAL BENEFITS: Discussing your experiences with Palliative Education and Care for the Homeless will allow us to help us to determine how to improve it for other clients, as well as, how to improve the care provided by similar palliative care teams.

CONFIDENTIALITY: This interview will be audio-recorded without information that could identify you as the person being interviewed, such as your name. We are audio recording the interview so that we do not lose any details. If you decide to participate, you are encouraged not to reveal any information that could identify yourself or others. If you do reveal identifying information, it will be changed in any reports that might be published from this study. These interviews will then be typed out and saved onto a secure device that will be kept in a locked cabinet. All audiotape recordings will be transcribed word for word (except for any identifying information, which will not be transcribed). Recordings will be destroyed once the transcribed information has been assessed for accuracy. The audio tape recordings and the transcripts will be assigned identification numbers, and will not be labeled with your name or other identifiers. The transcripts will be stored with and destroyed at the same time as the other study data. Only the research team will have access to the interviews and typed out copies of the interviews. These copies will be kept in the locked cabinet for 7 years after the study is complete at the office of the Inner City Health Associates and then will be destroyed. Access to the information recorded in the interviews will be limited only to the research team. Study data will be kept for 7 years after the completion of the study and then destroyed. You will have the option to consent to having quotations used from your interview in the final report from the study with or without your name or personal information. You do not need to consent to having your quotations used to participate in the interviews.

STUDY RESULTS: We will use the findings from this study to inform how people can best provide care at Palliative Education and Care for the Homeless and reduce the barriers that prevent people from receiving good palliative care. Once we finish the project there will be opportunities to educate the care team at Palliative Education and Care for the Homeless, as well as, other similar health care teams to help them provide the best care possible for future clients. In addition, we plan to present this information at conferences across Canada and internationally, in peer-reviewed journals and in news reports. If we are able to present our reports, we will ensure the confidentiality of your interview by removing any information that could potentially identify you. We will also only include your quotations if you have provided consent for us to do so, which will also be anonymous.

PARTICIPATION AND WITHDRAWAL:
As above, your participation in this study is voluntary. You may opt out of the study at any point by telling a member of the research team that you no longer want to participate. Choosing to withdraw from the study will not impact the care you or your family receive at Unity Health. You will be given the opportunity to review a printed copy of the interview transcript within one week from the interview. This is for you to potentially make any changes to the transcript, which would include correcting factual errors or retracting statements you do not want recorded. You may remove yourself from the research study up to two weeks from receiving the transcript. If you decline to review the transcript, you may withdraw up to two weeks from the date of our interview. If you choose to remove yourself and your interview from the study, all information you have provided during the interview will be

© 2026 Levesque AR et al. *JAMA Network Open*.

withdrawn from the study and destroyed. If you decide to participate, you can skip any questions you do not want to answer. There will be no effect on your relationship with the health care providers at Palliative Education and Care for the Homeless if you decide you would not like to participate in the study.

RESEARCH ETHICS BOARD CONTACT:
If you have any questions regarding your rights as a research participant, you may contact the Chair of the Unity Health Toronto Research Ethics Board at 416-864-6060 ext. 2557 during business hours.

Please contact Justine Baek (Page 1) if you have any further questions.

SIGNATURE PAGE:

The research study has been explained to me, and my questions have been answered to satisfaction. I have the right not to participate and the right to withdraw without affecting my relationship with the health care providers involved in this study. As well, the potential harms and benefits (if any) of participating in this research study have been explained to me.

I have been told that I have not waived my legal rights nor released the investigators, sponsors, or involved institutions from their legal and professional responsibilities. I know that I may ask now, or in the future, any questions I have about the study. I have been told that records relating to me and my care will be kept confidential and that no information will be disclosed without my permission unless required by law. I have been given sufficient time to read the above information.

1. I, consent to participate in this research study through both written and audio recordings.

Participant name (print)	*Participant signature*	*Date*	*Time*

2. I consent to have quotes from my interview without

any identifying information to be used in publications related to this research study.

Participant name (print)	*Participant signature*	*Date*	*Time*

3. I have explained to the above-named participant the nature and purpose, the potential benefits, and possible risks of participation in this study. All questions that have been raised about this study have been answered.

Name of person obtaining consent (print)	Position/Title of person obtaining consent (print)	Signature of person obtaining consent	Date	Time

© 2026 Levesque AR et al. *JAMA Network Open*.

eMethods 2. Interview Guide

Pre-Interview:

- Introduce self as being part of the team that is conducting a PEACH research study that they were interested in being a part of.
- Ensure they have reviewed and understand consent form:
    - Particularly that they understand the risks of participating, that their participation does not affect the care they receive from the team and that information provided is anonymous.
    - Ensure to mention that they can withdraw consent if they want to at any point in the study.
    - They can also decline to answer any interview questions or stop the interview if they feel uncomfortable.

Section 1: Introductions (5-7 minutes)

1. How did you connect with the PEACH team initially?
2. Who was helpful in getting you connected with Palliative Education and Care for the Homeless?
    a. Can you describe the experience?

Section 2: Benefits and Impact (20 min)

1. What have you been told about Palliative Education and Care for the Homeless and what this team's role is for you?
    a. Do you know why you have been receiving care from PEACH?
    b. Other than medical issues, has the PEACH team helped with any other concerns such as social or financial issues?
2. Do you feel PEACH is serving your needs right now? If yes or no, why is that the case?
    a. If PEACH has helped, in what ways has the team helped with your medical care?
    b. Has PEACH helped you with for non-medical or social needs such as transportation, finances or living conditions?
    c. Has PEACH worked with any of your healthcare teams or community supports (shelter staff, case workers) to address your needs?
3. How has the care you have received from PEACH differed, if at all, from care provided at other medical teams or clinics that you are involved with?
    a. Have you run into any challenges with other health care teams that PEACH has helped address?
    b. Has PEACH helped you overcome any other barriers in the system? What kinds?
4. If you were not served by Palliative Education and Care for the Homeless right now what do you think your health would be like? Why would that be the case?
    a. Without PEACH, what would your social situation be like? (i.e. housing, food security)
    b. How would you have addressed your needs if you did not have access to the PEACH service?
    c. Can you describe what your care would look like without PEACH? (or before PEACH?)

© 2026 Levesque AR et al. *JAMA Network Open*.

Section 3: Barriers or Areas of Improvement (10 minutes)

1. What would you change about the PEACH program if you could?
    a. Examples of aspects including referral process, making appointments, type of care received etc.
2. Do you feel that the Palliative Education and Care for the Homeless would be a good service for other people in a similar situation to you?
    a. Are there any reasons why it may not be good for these people?
    b. Can you provide an example?
3. Are there any concerns or health needs that have not been met by the PEACH team? Why is that the case?
    a. Is there anything you need right now?

Section 4: Final Thoughts (10 min)

1. What is the best thing about the PEACH team?
    a. How has care from the PEACH team affected how you live with your medical issues?
2. If you had a magic wand and could change anything about the PEACH team, what would you do?
3. Is there anything that we need to know?

© 2026 Levesque AR et al. *JAMA Network Open*.

eTable 1. Supplementary Quotes – Life Before PEACH

Sub-Theme	Participant Number	Quote
Hard Life	1	I didn’t go through all of the stages; I got like right away stage four metastasized already I have lung cancer and went to the liver and lung cancer I got 2 years 8 months of which were functional. A year ago, just about when they said that.
	1	To give you a li’l bit of background, my wife got ALS [amyotrophic lateral sclerosis] about 5 years ago, and that’s it, died, and after that I ended up homeless.
	1	Like it’s really hard for me to find somewhere to go. If I would be alone, I wouldn’t make it when I come back from chemo, chemo is hard, immunotherapy is hard. I have a hard time going to the bathroom, I wouldn’t be able to do my meals. So, I need a place like this like the [confidential] program. Alone in an apartment, I wouldn’t make it. It would have to be supported. And that would be impossible to find right?
	1	Interviewer: If you didn’t have access to PEACH, what do you think or how would you think you would’ve addressed your needs?<br><br>Participant: Oh, I would’ve given up. I would’ve given up after the wife passed. I-I- I- was really, really in a bad place, and having a cancer diagnosis is horrible. I would’ve given up for a 100% I would’ve given up for sure. No question about it I wouldn’t made it through.
	2	Just to get in this place and get a place, a lot of people wait years. I mean I’ve been in the shelter system for almost three years but still [unclear] people seven, ten years.
	2	If I don’t feel safe, if I don't I can say that if it wasn’t for my size or everything I didn’t feel safe, nobody will bother me physically okay, and I guess they just figure out [unclear] mentally, maybe that's what they want me to do, they want to drive me crazy, so they’ll keep it at Fred Victor, one time I did go a little crazy and almost tried to kill myself. After that I just thought, these guys aren’t going to control my life anymore and I when they get to me or whatever I would just make a joke or whatever.
	2	The staff there, my ex-wife, she made sure my ex-wife, even now I uh money from old age she gets $500 a month and I got a court order that was stolen out of my locker and everything and still [unclear] I had a copy of it and I was supposed to be $25 a month because the judge it was a lady and I thought I was dead because she looked like she was 75 year old but she was smart as a whip this judge and she uh, when my ex tried to say I didn’t pay 28,000 dollars well I paid 1,000 in checks by being a nice guy at the start I had an account together already I totally had to pay [unclear] and she told my three daughters you don’t see your Dad you're not in this family. Well, my two daughters they listened to that and my other daughter she said no, no, no I’m seeing Dad what you are talking about. She ended up dying and uh my ex didn’t even phone me about the funeral or something. My son found out on social media and stuff, he lives in Vancouver, he phoned me, he doesn’t usually phone me because we text every day I probably got a text on my phone right now I was just talking to him, and he said I phoned you dad because [Unclear] [she] died of an overdose and the funerals tomorrow and I know [Mom] probably didn’t want to tell you, so I went to the funeral and I think she was surprised I was there.

© 2026 Levesque AR et al. *JAMA Network Open*.

	2	[...] [My daughter] didn't care about that she wanted to see me and after 6 or 8 months I had a girlfriend, and she was being nice to [my daughter] and giving her some makeup and stuff and she wore makeup she was almost 16 then and she wore makeup when she was at home with us. Unbelievable singer she won the rising start competition at the exhibition out of 5000 kids, she did remax [sic] shows and everything and the people there were like oh my gosh I'm getting chills. She was singing Whitney Houston "I will always love you." and so good, this [unclear] God said she's wasting her life after doing all the drugs and took her up as an angel I think.
	2	A lot of my depression was around that, big time, and back then when I was in my depression I tried to kill myself. Other times doctors gave me pills to stop smoking, stop drugs and everything, I moved up to my dad's in Newmarket, and that 28 days of being clean and everything I guess these pills started taking the side effects and my doctor even said "who gave you these pills" well I guess it was the backup doctor, she said my backup doctor was a she [...] so this man doctor just gave me these pills [...] I took the pills [and] there was a bottle of bleach there and I must've drank a half a bottle of bleach and there was a knife there and I started stabbing myself.
	3	[...] the cancer was so widespread [...] it's completely in my upper body, spine, chest, ribs, the whole upper body, chest, clavicle, skull. It's on my bones, on my femur muscles, one of the worst [...] So all they were doing was looking to stall it and try and stop the cancer from growing, so they took all my testosterone out of me and [...] their goal I guess was to contain it , stop it as best they could and try and kill all the cancer that they could so these were where all my shots were coming in. They give me the three-month shot; they give you the Apalutamide for the pill. It's like a pill chemo because they told me I was too far gone for regular chemo and that right? So, they said we'll just try and control it with the other things
	3	[...] when I was at the other place-we always knew I was coming here or get a bed or stay in prison -so of course I took the bed, and I was just waiting to come here. So that was a different place altogether – ahh- I wasn't required to do any daily chores or anything like that and there was nothing for me to do and it was the middle of the winter, so I really wasn't much into going out [unclear] with all the help to get my ID.
	3	[...] when I was inside, I was only going to see the cancer clinic pretty much every week -and the doctor there said "I will make sure that I look after you and that I am going to make you better- right? He said that I promise that. But, when I went back to the institution everything that he had prescribed and done for me, they wouldn't allow it. Because, no, inside we… So, finally he went as far as to say- look either you're going to give him the proper medications - this guy's got cancer- and we want him on that and if you don't give it then were going to take this to some place where you're going to be having to explain it to a judge right? So, they snapped right out of it right away. But they didn't give any kind of palliative care in any way at all, nothing, if you ask for something, you're not going to get it.
	3	I was living in a housing kind of unit but I had to go, walk across to the health care every morning to get my meds so there's nurses there and the doctor would come in once a week- well , he didn't really know anything about cancer or anything like that- he was just look at what the cancer centre, clinic sent over and whatever and he would even ask for something and then the nurses would too and the people that in the pharmacy there said no – our policy is no.
	3	They had to give you pain relief and I was in a lot of pain and without any kind of long term or short cut throughs and stuff like that and they said and it was …it was my cancer doctor and a woman there and she said you leave this with me and I'll have this cleared up and the two of them got a hold of them , straightened them right up, scared the crap right out of them and then, and I got everything I needed after that – but no palliative care.

	3	Between them and here- both of them… so good, so kind both of them are so kind, it's amazing. I'm not used to that kind of kindness inside [prison] because they're always making things difficult for you.
	3	I came out [of prison] really worried about seeing people, thinking they know you're out of prison and that -you know, they don't know…but in your mind you don't know that and you're looking. I didn't even know how to use a phone, things like that. And a card- I didn't know how to get my ID and then slowly but surely, I'm learning now right?
	4	It was [the shelter], there was a doctor there, he said I had like three days to die, it was all this stuff. Just smart-ass doctor. Anyways it worried me, I got involved with them yes.
	6	I was in the hospital for two years and three months and I got out and I kept going back and forth back and forth multiple times and then they told me at Sunnybrook [Hospital] you've got two to three days to live and that was six years ago or whatever, so, you know, and it's just been really tough ever since. Um… due to the fact that, you know when they told me I didn't have long on earth I sort of prepared, started preparing myself you know. Then it didn't happen, so I let my guard down, you know, but you still sit, and you think, and you think, and you think. And you know now I'm to the point where I wish I would, because my lives just terrible. I can't do anything. I have no, I don't go out of this apartment, I don't have a life at all.
	6	Interviewer: And do you know why you've been receiving care from PEACH or why you were referred initially?<br><br>Participant: [...] Because at that time my social worker knew I wasn't going to make it on my own, I was barely making it, so… I was in pretty rough shape… so you know they've been there ever since.
	7	Right, everything happened so quickly, umm. I was diagnosed with on top of my COPD and emphysema and another lung disease that I have, on top of that I got diagnosed with multiple myeloma and I got a phone call… my intention all along for my end of life was going to be [Medical Assistance in Dying (MAID)] be it in ten years or in ten days, it didn't matter, it was going to be [MAID]. I got a call in December, and I thought I better move it, and that's what initially started my inquiries into how to apply for MAID services, was that I got a phone call saying that I had less than 6 months to live, and I thought I better move it, and see how fast I could get this ball rolling.
	7	I have this disease, non-tuberculosis mycobacterium avion, MAC, or it's got a couple of different acronyms for it [...] Anyway, the MAC wouldn't go away. There's a three-antibiotic treatment for MAC and after four years of being on this treatment for it, it was [apparent] it was never going to go away. I was frequently getting infections and had pneumonia and sometimes the antibiotics would work and sometimes they wouldn't, and I said to my lung specialist there has to be something to this, something is happening in there that's making this all do not work. So, she sent me for massive blood tests [...] [and] they found out I have multiple myeloma.
	8	I ended up getting really sick and ended up kind of in a coma, because I had pneumonia, and my oxygen levels dropped in my body, I basically couldn't walk and I was uh, I was in between my buildings and I couldn't walk anymore and my sister in-law has seen me and yeah next thing I know [...] I was waking up like three weeks later.
	8	My life is completely changed, I literally lost more than half my body weight. I had to learn how to walk again and learn how to eat again. In February I was just unloading trucks and now I'm walking with a walker.
	8	I've been doing the same job for 35 years and to stop doing that job after all those years, it's hard.

© 2026 Levesque AR et al. *JAMA Network Open*.

	9	I had a broken hip, and then I broke the other hip. So, I went into the hospital in [confidential]. The first hip I broke I was in for four and a half months, this time I was only in for two and a half months because I wanted to go home and I wasn't ready, I couldn't function at home [...] Like my apartment was dangerous for me. I'm a recovering addict, people are knocking on my door wanting to get high with me, it's really hard, I only have three months clean.
	9	Like there were eight kids in my family, and me and my brother were the only ones my dad beat, and we both became addicts. The others didn't even take a drink in their life or a cigarette. That's not a coincidence. That's our trauma. He beat me. Anyways, but I loved him. I loved him. And the only two things he would say to me was I was smart.
	9	My daughter when she woke up found me laying on the floor in poop and you know there was poop there. [unclear] [...] I did have a broken hip, I couldn't get up, I couldn't move and then when I broke this hip, the first hip I broke, I was doing so well, after six months I was able to go back to walking. And then I started to go downhill. What happened was my hip was infectious; the only way it could have happened was in that last surgical wound [...] they put a smith ball in there full of antibiotics then after six months they had to put a fake hip in. That was really a lot of recovery, ugh. And this one I thought I really thought I could go home, I couldn't, I couldn't cope I couldn't cope [...] Everyone in my building, it's a seniors building, everybody there is too poor or too infirm to help you know. But they want money.
	9	Well, I'm not homeless, but my home is precarious. It's dangerous for me [...] like one time a guy forced himself into my place and was beating on me and I was running down the hall. He grabbed me by the bottom of my head, hit [it] into the concrete floor and he was [...] He got out of jail in fifteen days, fifteen days.
	12	I took down sick in covid times, I got covid and liver cirrhosis.
	13	I was hit by a car in 2013, and I have a broken neck as well. I've got some bolts you know they bolted my neck to my spine and the pain from that is just outrageous, I'm in constant pain all day every day and I was getting such a low amount of pain help that it just wasn't working [...] and that doctor refused to upgrade [...] it was so hard to get an upgrade from him that well you become immune to the meds. You can only be on them for so long and then you need to get an upgrade or change [...]
Previously Encountered Barriers	1	[...] somebody with let's say lung cancer would be at home with one caregiver is not enough, not enough.
	2	You see one time I was in the hospital [...] all of a sudden, my health card was gone and the pills I had brought with me were gone from my end-table. Right in the hospital, the top drawer [...] I remember [the doctor] after the 5th or 6th day say, "so you did pretty good without your depression pills, you didn't have depression pills all week" and I said I was wondering why where my pills went, because you know I take my pills myself. And he said "I [don't] know what you're talking about [...] " And I said I let the nurses know the first day that my pills were missing so they should be giving me pills. "Well maybe you don't have depression."
	3	One of the reasons I was turned down… to go to [confidential] to be with my sister was because they didn't have proper palliative care there.
	3	You'd have hours where you would have to wait [...] I always had to run up to the hospital to see a doctor when I 'm having problems and then sit there [...] sometimes for four or 5 hours - then you go in and then it's another hour or so and just for a problem.

© 2026 Levesque AR et al. *JAMA Network Open*.

	3	I wouldn't be where I am right now, not a chance. [PEACH] are the ones that took the time to, nobody else thought about the power of getting my meds. The pharmacy didn't think about it. The [other] doctors looked at it, but they didn't think about it.
	3	I was living in a housing kind of unit, but I had to go walk across to the health care every morning to get my meds so there's nurses there and the doctor would come in once a week. Well, he didn't really know anything about cancer or anything like that. He [would] just look at what the cancer centre clinic sent over and [...] he would even ask for something and then the nurses would too and the people [...] in the pharmacy there said no – our policy is no.
	4	[...] there was a doctor there, he said I had like three days to die, it was all this stuff. Just smart-ass doctor.
	6	Interviewer: How do you think you might have addressed your needs if you didn't have access to PEACH?<br>Participant: I wouldn't be able to tell you that. Unless it was through this family doctor I have, I don't know. Because I had a social worker in his office, but she left. She and I had a really good relationship, other than that there's been nobody else that I've been able to talk to or team up with when I need help.
	8	[...] you know I've never been a doctor person [...]
	8	In all honesty, if PEACH wasn't coming here, I would not be going to any doctors. I'm not a travelling type of person. It's got nothing to do with wait times, if I have an appointment I have an appointment. And I'm not a walk-in person. Yea if they weren't coming here, I would definitely not be going anywhere. I'm the type of person to sit back and let whatever is going to happen is going to happen.
	8	I would still be living at my old place. Probably locked in my apartment, no way to breathe. Because I would not be going anywhere. I know [...] I know myself. Yeah, I wouldn't be going anywhere. [If it wasn't] for PEACH team coming here or coming to me and helping me, I would not be seeking help.
	8	I've worked with 4000 other people and basically, you're told to fuck off all day long, sorry for the language but that's exactly what happens when I'm out in the world. But [PEACH], they come to my place, and I don't know they're just so, so good. And I'm not used to that. Anytime I've walked into a walk-in clinic or anything like that they're just so, so impersonal. And with these people it's just like they're caring!
	9	Well, you know they tell me I have brain damage, I've had many concussions, many. And they can't tell. But the doctor told me, not from PEACH, that every time I use part of my brain dies, every time. He was an ass hole, I asked will it go the other way will I get smarter.
	12	I will be down, because some of the time I can't buy my medication, as I have to buy my medication myself, and I'm not working. So sometimes, as I said, I get various aches and somebody might sponsor me.
	13	[...] the previous doctor I had, and I still have I guess, he's not a pill doctor [...] And I'm in that much pain I just couldn't wait around; I had to move up you know. I was hit by a car in 2013, and I have a broken neck as well. I've got some bolts you know they bolted my neck to my spine and the pain from that is just outrageous, I'm in constant pain all day every day and I was getting such a low amount of pain help that it just wasn't working you know what I mean, and that doctor refused to upgrade [...] It was so hard to get an upgrade from him that well you become immune to the meds. You can only be on them for so long and then you need to get an upgrade or change you know [...] God bless him, he was a great doctor he really was he just didn't like working with the pills.

© 2026 Levesque AR et al. *JAMA Network Open*.

	14	I had a bladder infection I couldn't get rid of. They started writing prescriptions for that because I just wasn't getting to a bladder doctor.
	14	Interviewer: When you didn't have the PEACH team who would usually help you fill [disability forms] out?<br><br>Participant: I asked every doctor to have it, and everyone said they were going to do it, and no one ever did it
	14	Interviewer: have you been connected with psychiatry before or was this your first time you were able to get connected -<br>Participant: Oh yeah they [involuntarily admitted] me for four months in hospital, that's how connected with psychiatry I was [...] I've never had a psychiatrist speak with me before [...] in fact they wouldn't even look at me, I've never had one speak to me [...] I've been formed a bunch of times and I've never had anyone speak to me, they just look at me and walk away, then I can't go anywhere and I can't smoke and I can't, you know what I mean, I can't get any food and I can't get anything.
	14	I have a lot of doctors so someone's always catching something but like I said they're all disconnected so the PEACH team just sort of steps in and says yeah, we can just [do it]. They don't say "that's not my area" whereas all of my other doctors are kind of on their own focus.
	14	I have asked every fucking doctor I've ever had since they've been locking me up [...] and no one has ever even taken a look [at the disability form], but then they never do [...] I've asked every doctor, I've printed it off and handed it to people, nobody's ever helped me out.
	14	I got diagnosed with [the bladder infection] in [an] emergency room and he called in my prescription, and it just never made it to the pharmacy, like no pharmacy had it at all. And that was the beginning of the nightmare of even getting it treated for three months or so.
	14	Interviewer: How were you taking care of your mental health before the PEACH psychiatrist? Were there people helping you with that at all?<br>Participant: I was afraid to touch it at all because I was afraid of getting locked up in a hospital again if I opened my mouth, so I was afraid to say anything to anybody.
	14	Yeah, it's hard to talk to doctors, they have a certain idea in their head about your care and its sort of hard to bring up your own, you know what I mean.
Stigma	1	I kind of kept it to myself [...] It's hard to tell somebody "Hi I have lung cancer; can I go to your place and die?"
	3	I came out really worried about seeing people, thinking they know you're out of prison [...]
	9	Interviewer: If you don't mind me asking, do you feel like other doctors ever treated you differently because of your cocaine use.<br><br>Participant: Oh yeah, if they knew!
	13	Interviewer: Do you think people in the healthcare system, like doctors or nurses, have ever treated you differently because you use?<br>Participant: Well, let's just [...] you're frowned on you know what I mean. Pretty much everywhere [...] because they look at like if you weren't doing this you wouldn't be here.

© 2026 Levesque AR et al. *JAMA Network Open*.

	14	Interviewer: How were you taking care of your mental health before the PEACH psychiatrist? Were there people helping you with that at all?<br>Participant: I was afraid to touch it at all because I was afraid of getting locked up in a hospital again if I opened my mouth, so I was afraid to say anything to anybody.
Substance Use	4	I know I was a big drinker and I'm back to being a big drinker again, not really a big drinker I just drink a lot [...] my stomach is a problem like from drinking I've got like so many problems [...]
	4	I continued drinking really hard and I continue drinking really hard like I'm at three 26s a day and then some [...] I was just trying to get out of my mind, just to get away, and all I do is really hurt myself. Like my livers gone [...] [a] whole lotta stuff in my stomach likes all screwed right up [...]
	9	I'm a recovering addict, people are knocking on my door wanting to get high with me, it's really hard, I only have three months clean [...]
	9	I was addicted to cocaine, that's totally different. I never had problems, I had cancer three times, and I never had problems coming off of opiates ever. I don't like them; I don't like being low energy [...]
	9	I was sober for seven ears. I went to rehab, and I was sober for seven ears. I relapsed five years ago but I was sober for seven years.
	9	Childhood trauma. Like there were eight kids in my family, and me and my brother were the only ones my dad beat, and we both became addicts. The others didn't even take a drink in their life or a cigarette. That's not a coincidence. That's our trauma.
	9	[...] I think they would see past the addiction and knew who I was. You know, I was so unhappy [...] I didn't want to use but then when I was sober for seven years, I was so happy again.
	13	Interviewer: Do you feel like the help you get from the PEACH team helps you not have to go to the street?<br>Participant: That too, yes, very much so, I can't argue that. You can only take so much pain it just, it wins. All you can do at the most is maybe calm it for an hour or so you know what I mean [...] Because, considering where we are to go to the street is so easy you know, it's a given.

© 2026 Levesque AR et al. *JAMA Network Open*.

eTable 2. Supplementary Quotes – PEACH Patient Services

Theme	Sub-Theme	Participant Number	Quote
Outreach and Community Care	Care Coordination	1	Interviewer: Can you describe a little bit about what the PEACH team does for you?<br>Participant: Everything that is cancer related. From setting up the appointment to dealing with the doctors to getting the transportation, making sure people are coming with me that I'm well supported, everything. And excellently!
		1	PEACH is on everything. From transportation to having someone escort me [...] they're amazing through and through.
		2	They have increased my meds that are needed and brought me right to my appointments. [The Nurse Coordinator], with her purple car [...] Yeah, she's been driving me to my appointments,
		2	We went in her car. I'm a neat freak, I'm very clean, but we went to see the apartment and it was amazing. Nice wooden floors and a good size bathroom. So tomorrow I'm going to check on the furniture.
		3	[...] they offered each and every time I was going up to [the cancer hospital]– they had asked if I wanted them to come along.
		4	They just, the things they do, they go beyond themselves [...] They got me a nice hospital bed, they ease my pain, they help me see a liver specialist, they're helping me see all these specialists, they, it's just, its wild.
		7	I give kudos to the PEACH team for how quick they reacted to the initial conversation with sending over doctors, inquiring what kind of care I needed for PSWs, and things like that.
		7	They helped me with a walker, a wheelchair, put me in contact with the provincial program that got me my scooter. So yeah, as far as equipment that I could use to give me more mobility, because of the lung issues, yeah, they were very, very helpful. Takes a while when you're working with, especially when the government's involved I know that it takes a while, but yeah, they put me in the right direction and hooked me up with the proper people. I didn't get any run arounds.
		8	[The Nurse Coordinator and Health Navigator], they're more like a like a bridge to the doctors. You know if I have a problem I call [the Nurse Coordinator] and then she directs that call to wherever it should go.
		8	Yeah, my breathing has got so bad, like my lung function is basically down the pipe maybe 25% 30%. They helped me get back in touch with my respirologist, so I finally got in touch with her and I'm going to rehab for my lungs and for my body and I start next week.
		8	They've helped me [...] leave the house and run to the doctor's office. When I leave the house, I've only got three hours of Oxygen to take with me. So, everything is just like kind of balanced in time and now [I'm] currently stuck with three-foot leash because I'm relying on it all day long.

© 2026 Levesque AR et al. *JAMA Network Open*.

		8	I know for a fact that if I needed transportation, I can call PEACH and they would be here for me. That I know.
		8	I talk to [the Nurse Coordinator] at least once a week. Yeah, even when I don't really need anything, I send her a message just to let her know everything is okay right now [...] I usually text her you know just to say good morning.
		8	If I call [the Nurse Coordinator] today, I know [a PEACH doctor] will be there in the next day and a half. Or at least if I need to talk to [the doctor], I know he's calling me that afternoon.
		9	I wanted to go home, and I wasn't ready, I couldn't function at home, so finally PEACH found this place for me, this hospice, and I'm so happy.
		9	They got me a hospital bed, they got me a walker, if I don't have the money they'll get it. They even got me a new set of sheets for my hospital bed. I love them, and that personal care worker [...] I don't know how to do without her. She does my laundry, she comes four days a week and on Thursdays she's there for four hours because she does my laundry, and she goes to the store for me [...] you know I'm so grateful to her.
		9	All I do, something happens I make a phone call and they're there. The nurse, oh my god, the nurse is so good.
		10	I'd say for instance it's not [that I call her] my boss because [the Health Navigator] bosses me around but no, she doesn't boss me around, but she's strong shown so much good leadership …she's understanding, she'll listen….even when she's late or something, she's going to call you to either reschedule or do something. She'll get it done, those are some things I like about her, you know.
		10	They give me nurse to check me, you know, all of that.
		12	[They help me with] my medication, sometimes when I can't afford it, and then they give me gift cards to purchase things. Food. Because they don't allow me to work, so they give me discounts [...] and they even volunteer to come and go and get it for me and [They are] always calling, coming to see me, sitting there and talk with me and all of those. [The Health Navigator] always called, she's encouraging. She finds the doctors to send to me. She finds whatever I talk to her about, she will work on it. And she helped me a lot in the Peach team.
		12	They offer transportation to wherever I want to go. Sometimes it's just to the hospital so I tell them I can just jump on the bus [...] but they always insist to come and help.
		12	They will make [the] appointment. They will [...] [say] "okay, the doctor wants to come and see you tomorrow."
		13	I'm getting things done now, you know what I mean. I guess everybody wasn't wanting to be that outright responsible [...] and the PEACH team stepped in and looked me over [...] If it wasn't for them, I wouldn't be nowhere near where I am
		13	They got my appointments set up, directing me to the right doctors [...] Again, I think these guys are wizards, seriously I do.

© 2026 Levesque AR et al. *JAMA Network Open*.

		13	[...] they come right to me and tell me, you know what I mean, and it's not done through third parties or over the phone
		14	The MRI once was scheduled for 2am and they were there and confirmed it was at 2am at [the hospital] [...] They'll call my specialist and check on things.
		14	I have a lot of doctors so someone's always catching something but like I said they're all disconnected so the PEACH team just sort of steps in and says yeah, we can just [do it]. They don't say "that's not my area" whereas all of my other doctors are kind of on their own focus.
		14	You know they call me to remind me about appointments that have been made three months prior because they're trying to get [me] into something that's very popular [...] Yeah, they'll call me and be like "hey remember that CT scan that was scheduled," or whatever, that ultrasound, it's today or its tomorrow, get ready.
	Collaboration with Other Agencies	2	[The shelter nurses] will phone the PEACH team if I have meds that [are] coming up.
		3	PEACH has to be involved with [the house staff]- they call them up and they say okay he needs this and that, right?
		4	I've got a counsellor there and I guess that they you know they get a long, yeah [the PEACH team] work with everybody as far as I'm concerned.
		6	I think they've gotten in touch with my other doctor when need be.
		7	Initially it started with me trying to find out about applying for [Medical Assistance in Dying], and I was directed to initially speak with [the home care coordinator]. Anyways, that's how it all got started [with PEACH].
		7	They helped me with a walker, a wheelchair, put me in contact with the provincial program that got me my scooter. So yeah, as far as equipment that I could use to give me more mobility, because of the lung issues, yeah, they were very, very helpful. Takes a while when you're working with, especially when the government's involved I know that it takes a while, but yeah, they put me in the right direction and hooked me up with the proper people. I didn't get any run arounds.
		7	One of the [PEACH] doctors that came to see me was familiar with my oncologist and had communications with her before, and that helped.
		9	I wanted to go home, and I wasn't ready, I couldn't function at home, so finally PEACH found this place for me, this hospice, and I'm so happy [...] And then three of the doctors here [at the hospice] [...] had been to my place with PEACH.

© 2026 Levesque AR et al. *JAMA Network Open*.

		10	So [the PEACH doctor] [...] brought up this idea of hospice services. I brushed it off initially. I said no, I don't like the idea of this hospice thing. I don't see myself as the end of my life [...] her concern was that she can't carry the workload alone…she been [to my house] so many times… she needs her colleagues to help me if she she's not around and she thinks from the results she been having checking on me constantly I'm doing okay, but more is needed for pain management. I've been abandoned and it's the end of life for me [...] So, she set up, she did everything [...] she called, and she said everything has been okay and it has been approved and [I'm] going to get out of my place and come [to the hospice].
		12	Yeah, I think the other day, I don't remember the [PEACH] doctor[s] name, but [...] she was on the phone with the family doctor, talking to her, asking her for some result.
		13	Yeah, I have a counselor that looks out for me, and they converse a lot [with the PEACH team] [...] Yeah, it's great because they have this connection and the worker, I have here is also [...] pretty good too and she comes to me with any info. Anything I have she's right here [...]
		14	They'll call my specialist and check on things.
	Connecting With PEACH	1	[The Health Navigator] called me on a Monday and told me that they would be assisting me in dying [...] I didn't go through all of the stages, I got like right away stage four metastasized [...] I have lung cancer, and [it] went to the liver [...] I got 2 years, 8 months of which were functional. [It was] a year ago just about when they said that. And the team helped me through that when I needed a hand.
		2	Participant: I think it was through [the Nurse Coordinator] when I was at [the other shelter] and she was trying to get me into a bed because it was all cots there and you can't really sleep at all because once it's besides each other the whole place wakes up. So, she tried to get me in [this shelter] and she did. And after that she brought me to a couple of appointments for my cancer and that too and then she ended up helping me get a place.<br>Interviewer: That's awesome. Do you remember how she actually started to follow you, who connected you with her?<br>Participant: It was somebody in [the other shelter] [...] said we know somebody who could probably help you and I guess he'd dealt with them before. He told me they're great at helping out, getting the meds you need in case it gets mixed up or this and that
		3	I believe that was done through [...] maybe the last house where I stay[ed] and possibly because PEACH was dealing with [the shelter's] pharmacy.
		4	Participant: I was in [the shelter], I got involved through them. It was [the shelter], there was a doctor there, he said I had like three days to die, it was all this stuff. Just smart-ass doctor. Anyways it worried me, I got involved with them yes.<br>Interviewer: So, was it that doctor who first connected you or was it someone else?<br>Participant: It was the whole place, yeah it was the supervisor, yeah, she cares about us too<br>Interviewer: And did they first see you at [that shelter]?<br>Participant: Yes, this did
		4	The only thing is, I don't really know how I got the connection, but I did, I really did. And that was beautiful to. You know I don't even use these words normally, beautiful [...] It's just I

© 2026 Levesque AR et al. *JAMA Network Open*.

			don't even know how we got connected but I'm happy I did, because it's all been positive, it's all been good.
		6	Initially it was I believe a social worker I had at the time who was being promoted. I was in a real tough position at that time, you know they told me I was going to die and all this other stuff, and he brought by the PEACH team to meet me, and [they've] been there ever since. That would've been I think almost 6 years ago [...] At that time my social worker knew I wasn't going to make it on my own, I was barely making it, so I was in pretty rough shape. So, you know they've been there ever since.
		7	Initially it started with me trying to find out about applying for MAID [Medical Assistance in Dying], and I was directed to initially speak with [the home care coordinator] [...] Anyways, that's how it all got started [...] Everything happened so quickly. I was diagnosed with on top of my COPD and emphysema and another lung disease that I have on top of that I got diagnosed with multiple myeloma… my intention all along for my end of life was going to be [MAID] [clarified after that client meant MAID] be it in ten years or in ten days, it didn't matter. I got a call in December, and I thought I better move it, and that's what initially started my inquiries into how to apply for MAID services, was that I got a phone call saying that I had less than six months to live, and I thought I better move it, and see how fast I could get this ball rolling. It all got mixed up along the way because my oncologist had a conference with my pulmonary care doctor, my specialist, and between the two of them they decided to offer up um a relatively new chemo treatment. You know this is already after I had started the ball rolling for MAID, so I didn't know what was going to happen, all of a sudden I have all of these things coming from everywhere and I didn't know if the chemo was going to take or work, so I just followed along with the program through PEACH and I have to admit that I give kudos to the PEACH team for how quick they reacted to the initial conversation with sending over doctors, inquiring what kind of care I needed for PSWs, and things like that to those services mostly provided by Home Care.
		8	Actually, my sister-in-law got [me] connected. She knew somebody, a nurse from the PEACH team. And so, I ended up getting really sick and ended up kind of in a coma, because I had pneumonia, and my oxygen levels dropped in my body. I basically couldn't walk, and I was in between my buildings, and I couldn't walk anymore, and my sister in-law [had] seen me and yeah next thing I know [...] I was waking up like three weeks later. I got out of the hospital and my sister-in-law she knew one of the nurses through a dog rescue they did together, and she sort of just mentioned my name and what happened to me, and they ended up hooking me up with PEACH and in all honestly thank God they did because PEACH has helped me greatly so far. I can't imagine where I'd be this far ahead without PEACH.
		9	Participant: I've been with PEACH ever since the doctor told me I was palliative and I'm so grateful. They give me 18 months to live, I bypassed that long ago [...] They knocked on my door, two doctors introduced themselves [...]<br><br>Interviewer: Do you remember who it was that connected you to PEACH?<br>Participant: A doctor, from [the hospital] got in touch with them I guess [because of] the label palliative [...] that was when I had a broken hip.

© 2026 Levesque AR et al. *JAMA Network Open*.

		10	[...] middle of February after my discharge from the hospital, I followed up [...] [someone from the] social work division I think [...] she's the buddy of [the Health Navigator] [...] [she] was the one who showed up first. It was quite [a] cold that day I remember. And then, basically, she started assigning medical staff. I had been given the medication, heart medication, obviously I needed new [refills]. So, she came in and she assigned one doctor. When [the doctor] showed up [...] she told me she was going to get refills for my medications [...] In a week or so, I got refills to my place, thankfully. So, we took it from there. And then, so let's see. About two weeks after meeting her [I] started to see some improvement [...] The first time I tried to reschedule, I got a really bad impression of her. I was wrong [...] A very good first impression of [the Health Navigator].
		11	Actually, they visit[ed] me, at the hospital [where] I [got] the surgery.
		12	I was in the hospital, and somebody introduced the peach team to me [...] I took down sick in covid times I got covid and Liver cirrhosis.
		13	Participant: Through the medical staff at St. Mike's. I was in there because of urinating problems, and I ended up referred to them [...] They found I have cancer in the throat and from there it kind of proceeded [...]<br><br>Interviewer: Okay, and do you remember who in the hospital connected you to the PEACH team? Participant: One of the shift nursing staff [...] became friends with me and basically said he was going to do this [...] I had no reference for the PEACH team or anything, things were moving really fast for me and I felt like things were just going to slow and I was starting to get worried and what not because of the throat cancer so I sort of progressed to saying okay let's check them because maybe, and things did turn around [...] They showed up right at the hospital [...] Yeah, it was a nice feeling [...] I was able to relax a bit, that's a big help when it comes to pain, you can relax a bit, and it takes some of that pain away. Interviewer: How soon after you went home did the PEACH team come and see you?<br>Participant: The next day.
		14	[...] Someone referred me from [the cancer hospital] [...] because I'm like a palliative sort of situation [...] They called me and they came out here, the first person they sent was a psychiatrist [...] I didn't even know, so when I was connected it was a huge surprise.
	Ease of Access	1	Every time I have the slightest problem I call [the Health Navigator] right away. And she really fixes it or points me in the right direction.
		1	[The Nurse Coordinator], like when I come to her with a problem, they drop everything and gets right to it. These are the people that I have that I deal with, and they are all really, really good.
		1	We speak every three weeks [...] I haven't had like a need for any weekend stuff. Oh, but I'm sure, if I call [them] like 24 hours a day [...] even if I'm just said, [they're] there [...] 100%.
		1	Interviewer: [...] What is the best thing about the PEACH program?<br>Participant: That they are around. That they are always present.

© 2026 Levesque AR et al. *JAMA Network Open*.

		2	They [said] just phone me if you get [depressed], and [the Nurse Coordinator] said doesn't matter the time, just call me.
		3	[The PEACH doctor is] coming in [...] every week, and then I told him don't come in every week anymore, you have other things to do, unless I'm feeling bad, or something changes, and I'll call you in advance and then you can come.
		3	With PEACH you know when they're coming. They will adjust their schedule for you or vice versa and [it's] so much easier with PEACH.
		3	I got all the numbers. I just make a call to PEACH. Yes, they're so friendly, yes. "Who do you need to see? Right?" And I say "Okay, can I see this person, can I get some more of this," or "can I change that," or "I don't feel well." "Okay we'll send the doctor in or whatever." Or I need more of the Pamper things there [...] they'll go to work on that. Anything I need, I call and they're right there.
		3	When they call its always so pleasant. If I call them, same thing. Call anytime they say, that makes you feel good knowing that they're available all the time. I can call 8 o'clock at night and if they're gone, I can just leave a message. They call me first thing in the morning, "What's the problem and what do you need. Okay?" It doesn't get any better than that.
		4	They're right there all the time. They're right there. See I'm on methadone too and they make sure I have that also. Yeah, they're right there for me and you know it's wonderful. It really is a wonderful thing.
		4	They're there for you, if you need the help, they're there to help you [...] they'll always be there too. They're great people.
		6	When I need some medical attention or something, they're there. And I have a family doctor who, you know, is one of these guys who works two days a week and is never in the office and like now he's gone till the end of December, but I've always been really [reliant] on PEACH. If [it's] not my [main PEACH] doctor, then somebody else will jump in.
		6	I know they're there for me when I need them, anything medical or anything else, there's always help somewhere.
		7	If I have a problem I call [the Nurse Coordinator] and then she directs that call to wherever it should go [...] and then just makes my life so much easier.
		7	I talk to [the Nurse Coordinator] at least once a week. Yeah, even when I don't really need anything, I send her a message just to let her know everything is okay right now.
		7	I'm not feeling well then [the PEACH doctor] will come, he'll be there the next day.
		8	If I call [the Nurse Coordinator] today, I know [a PEACH doctor] will be there in the next day and a half. Or at least if I need to talk to [the doctor], I know he's calling me that afternoon
		9	All I do, something happens, I make a phone call and they're there.
		11	That's easier for me if they see me at home.

© 2026 Levesque AR et al. *JAMA Network Open*.

		12	Sometimes when I'm sick, they're the only one I can call, and they will be there for me in a jiff.
		12	They're doing a wonderful job. And always communicate. And if you call and you don't [get] a hold, they will call you back soon.
		13	[...] in this time and age, I've never I don't hear of any docs who still house call [...] it speeds things up and things are done more at their discretion, you know what I mean.
		13	Interviewer: [Do] you feel like if you need them, you can get a hold of them, you can get them in? Participant: Yeah within 24 hours I'll have one right here.
		14	I've gone to the PEACH team for like naproxen before, they're good for that, sort of filling in [...]
		14	They're really good. They call me back right away when I need anything. If it's by text or by phone. I think they're not on so much on evenings and weekends but that hasn't been a problem for me yet.
		14	Yeah, I missed one [appointment] once. I went up to a volunteer position they called me in for and then I got [a] phone call as soon as I got there "hey I'm at your door." Whoops, so that was my fault. They called me the day before and everything, I just woke up and you know what I mean.
	Home Care	1	I'm in a [shelter] program, I don't like really to get around. It's a big place, there is a lot of people here. But [PEACH], they go through all the red tape and come right to my room.
		1	There is no treatment for ALS and all that and from that point of view we knew we would benefit and somebody with let's say lung cancer would be at home with one caregiver is not enough [...] PEACH would've been great there too.
		3	[...] one of the [PEACH] workers comes in and does my vitals and everything all the time and we talk and see if there's any problems and so she comes every week and my doctor's right there for me for anything I need.
		4	I've got a nurse that comes to see me three times a week. And it's just like they look after me so well, you know. Like, I've got bad [pain] in my legs and my back, I'm hunched over, and stuff and it really hurts. They just they help me with everyday life [...] because it's so hard to adjust sometimes. You just get so irritated, you just go "ahh" you just want to pull your hairs out and you know they just kind of ease all that for me. They come and see me too, the doctors actually come and see me, yeah, they reach out and they come and see me and that's amazing.
		7	I have to admit that I give kudos to the PEACH team for how quick they reacted to the initial conversation with sending over doctors, inquiring what kind of care I needed for PSWs, and things like that to those services mostly provided by Home Care.
		7	Like I said, this [Home Care Coordinator for PEACH] she knew everything. You needed something done she was the lady to call
		8	[...] they've helped me out greatly like you know I have doctors come to my house [...]

© 2026 Levesque AR et al. *JAMA Network Open*.

		8	In all honesty, if PEACH wasn't coming here, I would not be going to any doctors. I'm not a travelling type of person. It's got nothing to do with wait times, if I have an appointment I have an appointment. And I'm not a walk-in person. Yea if they weren't coming here, I would definitely not be going anywhere.
		8	To let them in my house, it was an [adjustment period], yes. But I'm just not that type of person, but so far, it's been positive for me.
		9	[...] and that personal care worker I don't know how to do without her. She does my laundry, she comes four days a week and on Thursdays she's there for four hours because she does my laundry, and she goes to the store for me [...] you know I'm so grateful to her.
		11	Interviewer: Do the peach team help with things related to the cancer?<br>Participant: actually, they yeah, they give me nurse to check me, you know, all of that.
		11	Interviewer: is it nice having a doctor come to your home instead of one you have to go to?<br>Participant: that's easier for me if they see me at home
		12	The PEACH team is a team that come in at home visits and they introduce themselves to me and started to talk. They tell me everything, encouraging me, talking to me, trying to tell me information about me, and then they encourage me about being active and not being sad. So, they always let me laugh and not feeling down or upset, even if I'm upset, they come to see me. They would encourage me that they're there with me for a good while, talking to me, let me smile, and leaving me happy.
		13	Participant: [...] back in those ages they did, there was still such a thing as house calls. Now that's wiped out. But yeah, in this time and age I've never I don't hear of any docs who still house call.<br>Interviewer: Does it make a big difference?<br>Participant: It sure does, it sure does, again it speeds things up and things are done more at their discretion you know what I mean
		14	I'm really [...] all over the place and it was good for me to not have to worry about getting out of the house, about getting money, and about being presentable in public
		14	I've asked every doctor, I've printed it off and handed it to people, nobody's ever helped me out and the PEACH team actually, the PEACH team actually did. He came out to my home and filled it out by hand right in front of me.
		14	Interviewer: What is the best thing about the PEACH team?<br>Participant: That they come to my house
Medical Needs	*Harm Reduction*	2	The methadone. They doubled that. It was a half a pill before, now it's a full pill. That was more for drugs, for uh people go to the methadone clinic for it, and they have a pill for it for me. Definitely have to give them credit for that.
		4	They're right there, see I'm on methadone too and they make sure I have that also.

© 2026 Levesque AR et al. *JAMA Network Open*.

		9	PEACH found this place for me, this hospice, and I'm so happy. Like my apartment was dangerous for me. I'm a recovering addict, people are knocking on my door wanting to get high with me, it's really hard, I only have three months clean
		9	Interviewer: Do you feel like the PEACH team ever treated you differently because of your cocaine use?<br><br>Participant: No! One time, the nurse from PEACH, she came there was a crack pipe in the ash tray [...]
		9	Interviewer: Any other kinds of people you feel PEACH would be good for?<br><br>Participant: Well homeless people, homeless people. People who are struggling to come off of a drug. Especially for women. Men have many, many resources out there. Not women, not women.
		9	I don't feel like they're working for me you know, they're giving me a service. I think they're my friends. And my nurses, you know I can talk to them about my addiction, even if I used an hour before I would still tell them.
		13	Interviewer: How would you have addressed your needs if you didn't have the PEACH team? For pain and things like that?<br><br>Participant: I would've gone to the street.<br><br>Interviewer: Do you feel like the help you get from the PEACH team helps you not have to go to the street?<br><br>Participant: That too, yes, very much so, I can't argue that. You can only take so much pain it just, it wins. All you can do at the most is maybe calm it for an hour or so you know what I mean. And I haven't had to as of yet and with the PEACH guys I don't think I'll ever have to [...] Because, considering where we are to go to the street is so easy you know, it's a given. And I can still say no to all that stupidness.
		13	[...] since I met them, I have to say [my drug use has] cut down some [...] Yeah it really has cut down some.
		13	Interviewer: You said sometimes you feel like you've been frowned upon [for drug use], do you feel like the peach team has ever frowned on you? Participant: Not even once, I have no sense or indication at all as far as that goes. They took me as I am and just that.
	Medical Supplies	3	They even got me the thing here, because I had trouble swallowing my pills and everything like that and my food coming down. So, they got me this [wedge pillow] so that I could be up like this at night [...] then I'm up like this so I'm not getting blocked on my foods and my stuff.
		3	Were you familiar at all that I had to use a diaper? Okay, they paid for that and got me that.
		3	[...] these things work really good plus they gave me special things to put underneath too.

© 2026 Levesque AR et al. *JAMA Network Open*.

		3	I got all the numbers. I just make a call to PEACH. Yes, they're so friendly, yes. "Who do you need to see? Right?" And I say "Okay, can I see this person, can I get some more of this," or "can I change that," or "I don't feel well." "Okay we'll send the doctor in or whatever." Or I need more of the Pamper things there [...] they'll go to work on that. Anything I need, I call and they're right there.
		4	They just, the things they do, they go beyond themselves [...] They got me a nice hospital bed [...]
		4	[...] they did everything, they even got me on everything, like the hospital bed, my back is screwed up and they got me a nice bed [...]
		4	Sometimes I don't even have a clue what's the matter with me and they tell me, like "you need a pair of socks." You know those socks that tighten everything up? My blood doesn't flow right. They went you need a pair of those things, so they went out and they got in touch with the nurse, the nurse got those things for me.
		7	They helped me with a walker, a wheelchair, put me in contact with the provincial program that got me my scooter. So yeah, as far as equipment that I could use to give me more mobility, because of the lung issues, yeah, they were very, very helpful. Takes a while when you're working with, especially when the government's involved I know that it takes a while, but yeah, they put me in the right direction and hooked me up with the proper people. I didn't get any run arounds.
		9	They got me a hospital bed, they got me a walker, if I don't have the money they'll get it. They even got me a new set of sheets for my hospital bed.
		9	They send me diapers and they send me my colostomy supplies at home [...] What was happening with the first nurse I had is I went 11 days without colostomy supplies and I couldn't eat and then the next time was six days. The new nurse I had [through PEACH], I get them before I need them.
	Pain and Symptom Management	1	[...] the doctors that come here are great for the meds. I'm in no pain and I'm comfortable.
		2	P: [Quetiapine], a sleeping pill. They took me down to 400 and I said that still doesn't get you to sleep right away so they added the 50.<br>Interviewer: So, the PEACH team was helping you with your sleep?<br>Participant: Yeah, they did.<br>Interviewer: Are there any other symptoms that the PEACH team helped address with you<br>Participant: Um, with the morphine pill they did.

© 2026 Levesque AR et al. *JAMA Network Open*.

		3	Basically [...] they had to change my drugs, because I was getting sick every single morning. And this is the biggest thing that they did [...] they talked about it, they worked with it a little bit, they found the right set up for me. I was taking something like 23 pills in the morning [...] and then I was sick every morning and it was from all the pills and that right away and my stomach wouldn't handle it. They said eat first and that was even worse, cause even if I had dry toast or whatever like they said I just was throwing up and it was making me sick all day, so they changed my meds [...] throughout the day, which is I can handle them all now, and they cut back on some that I needed. I wanted to cut back on some and they did the thing, and they did trial and error, and they found the right one and I haven't been sick in, God I think one time I got sick in the morning in the last 2 months.
		3	I haven't had to use a walker because I was falling down at the last place a few months ago before I came here. I was in the bed most of the time, I was sick, I wasn't eating, my weight went down, I was falling down even [...] And all that changed.
		3	[...] making sure I wasn't sick anymore. When I was having the problem with my bowels and things like that, of course I brought it up to the other doctors, but they didn't really have much to say about it. "Well, that's interesting," yeah, that's interesting all right (laughing). Yea, I poop in the bed. Nope the doctor here – he got me fixed up there so that hasn't happened now in close to a couple months now.
		3	I wasn't being able to eat food in the morning and I was sick every morning so of course they changed that so I could, so yeah that's the biggest thing.
		3	I was still in pain in the other place and then when I came here, I was and now they have me regulated properly and even now I've even cut back on my, the one opiate [...] I have maybe taken one or two in the last couple of months, and I used to take 5-6 a day.
		4	they gave me some medication to help me through what like I struggle and all that and like yeah, they just like not a lot where [it's] in my head it's just like enough where I can be like comfortable, you know. Not high or reckless really, just comfortable.
		4	[...] they help me with my stomach, my stomach is a problem like from drinking. I've got like so many problems there [...]
		4	Like, I've got bad [pain] in my legs and my back, I'm hunched over, and stuff and it really hurts. They just they help me with everyday life [...] because it's so hard to adjust sometimes. You just get so irritated, you just go "ahh" you just want to pull your hairs out and you know they just kind of ease all that for me.
		4	Interviewer: And if you were not served by PEACH right now, what do you think your health would be like?<br><br>Participant: I think I'd be really hurting, for sure, I'd be really hurting.
		6	We were trying, man, you know different medications. You know the problem with pulmonary functions is that a lot of the medications that are out there have a chance of uh making those symptoms worse and the gamble that I would have to take, is it worth it to take this medication if it makes it worse. Is it worth it?

© 2026 Levesque AR et al. *JAMA Network Open*.

		8	Yeah, my breathing has got so bad, like my lung function is basically down the pipe maybe 25% 30%. They helped me get back in touch with my respirologist, so I finally got in touch with her and I'm going to rehab for my lungs and for my body and I start next week.
		9	And then when I broke the second hip and the pain, I had so much pain. Now I'm pain free [...] like they had different rules than other doctors. I have very high tolerance [...] and they've done the best job I've ever had. I've had [pain] in my back for a long time and no one has handled my pain better than PEACH.
		9	You know, I couldn't do that before but now I feel like I'm living again. Plus, I had gone off all of my medications [...] I was even thinking about MAID, I'm just so tired all the time and in pain, I just wanted to have a final sleep you know. Not now, now I want to live.
		9	[...] they took my pain away, and when they got me on the right pain medication, it was a new me.
		10	[The PEACH doctor] told me she was going to get refills for my medications. It was good for my case, thankfully. In a week or so, I got refills to my place. Thankfully, so we took it from there [...] About two weeks after meeting her [I] started to see some improvement. So about three weeks after that time she showed up at that time [...] she said "[you were] in a very bad shape when I first saw you" and I could sense her feeling, [I was in] more pain [...] Oh yes pain, yes pain all over, the categorization of it was nerve pain. And that was terrible.
		11	Yeah, they give me some medication, no constipation you know?
		11	Actually, all the time they are asking me how I'm feeling but I'm okay you know.
		13	I got progression to levels where I could heal, you know what I mean so yeah definitely. If it wasn't for them, I'd be nowhere near where I am [...] Well because I was lying in the bed crying all day long because of the pain and now I can actually sit here and talk to you. Three to four months ago I wouldn't have been able to do that.
		13	[...] the previous doctor I had, and I still have I guess, he's not a pill doctor [...] And I'm in that much pain I just couldn't wait around; I had to move up you know. I was hit by a car in 2013, and I have a broken neck as well. I've got some bolts you know they bolted my neck to my spine and the pain from that is just outrageous, I'm in constant pain all day every day and I was getting such a low amount of pain help that it just wasn't working you know what I mean, and that doctor refused to upgrade [...] It was so hard to get an upgrade from him that well you become immune to the meds. You can only be on them for so long and then you need to get an upgrade or change you know [...] God bless him, he was a great doctor he really was he just didn't like working with the pills. But the PEACH team [...] they're not keeping them down as much as possible too, any agreement we make is done right away, these guys they just they don't sit around thinking about it they just do it you know, they get it done. Which again I'm also so thankful for man, I wouldn't be anywhere near where I am without them, they're a god send to anybody I think.
		14	[...] I've gone to the PEACH team for like naproxen before, they're good for that, sort of filling in [...]

	Primary Care	1	Last week I had a bad reaction to the chemo treatment, and I had a leg, my left leg started getting bigger and bigger and bigger and bigger and like right away they adjusted all the meds [...]
		1	They didn’t know if my leg thing was caused by immunotherapy or maybe a side effect but now the doctor came yesterday and already, I’m back so fast.
		3	[...] it was PEACH that got me better right. On top of that my diabetes that I had, I was on four diabetic pills a day and I’m on one now, but I don’t need any. They said I cured myself. Just from exercise and proper diet.
		3	When I had motor skill problems, they would work on that what they thought that it was [...] I wouldn’t be sitting up like this going and exercising, I’d still be laying on my bed sleeping most of the day and night.
		4	Sometimes I don’t even have a clue what’s the matter with me and they tell me, like “you need a pair of socks.” You know those socks that tighten everything up? My blood doesn’t flow right. They went you need a pair of those things, so they went out and they got in touch with the nurse, the nurse got those things for me.
		8	[...] the PEACH team got me [...] on a nutrition diet, I guess. Make sure that I’m putting enough calories in and stuff. So, it's good, they like make sure I get like Boost and stuff delivered to the house.
		8	I’ve learned so much from PEACH just because I learned how to eat, everything I eat it, it, it affects the way that I breathe. And yeah, I’ve learnt a lot, it’s given me a lot to think about and a lot to do. Which I appreciate that.
		10	They gave me a complete overhaul [...] This came up recently about COVID and meningitis vaccines [...] as to whether my mom took me for any vaccinations for that I didn’t know do I have to bother myself for that…and some of the doctors did find out.
		12	Sometimes when I'm sick, they're the only one I can call, and they will be there for me in a jiff.
		12	They give you information. They test you; they tell you things. They just don't. For instance, the doctors just don't come and just tell you’re this and you’re that and you are sick, no they talk to you.
		13	I guess everybody wasn’t wanting to be that outright responsible you know what I mean, and the PEACH team stepped in and looked me over which is I guess what they do
		13	I’ve got prostate [problems], and my urine ended up blocking my kidneys and that was getting to the point where I was in a very large danger zone, so they got me to a point where I was stable. I’m wearing a catheter right now and I should get it out in about a month.
		14	[...] I had a bladder infection I couldn’t get rid of. They started writing prescriptions for that because I just wasn’t getting to a bladder doctor.
		14	I’ve gone to the PEACH team for like naproxen before, they’re good for that, sort of filling in [...]

© 2026 Levesque AR et al. *JAMA Network Open*.

	Psychiatry/ Mental Health	1	Like the [person] I like have close contact with most of the time is [the PEACH psychiatrist], he is good for my brain.
		1	There's also the subsequent fallout from the moment that you know you're going to die right? The mental, the everything. They've taken care of everything.
		2	They've definitely helped me with my medications and my depression and really, I'm noticing good support. They've got my back maybe I should say.
		8	They've helped me out greatly like you know I have doctors come to my house, you know, and they've helped me set up like my meeting with my [psychiatrist].
		8	They got me hooked up with a [psychiatrist] which is okay. I never thought I would have to deal with [this], but it was something I really need and I'm actually surviving because of it, and without PEACH, I can honestly say I wouldn't be talking to this person.
		8	Interviewer: how often do you consult with your psychiatrist<br>Participant: Once every three weeks [...] At first it was every two weeks which I, I really needed and then now it switched to every three weeks [...]
		9	They talk with my psychiatrist who is with PEACH, he was here yesterday [at the hospice] to see me [...] He was here yesterday, and I see him every second Thursday.
		9	[...] I'd like to talk to you a minute about the psychiatrist. He doesn't have privileges here [at the hospice], I'm not sure. He comes to see me still and he, he gets to my core of the traumas I have, and I trust him enough to tell him. Because I had a hard time ever [trusting]. All of us have some secrets and [they] should stay there. Unless you have a good psychiatrist like me that you can talk to.
		12	[...] there's a lot of different doctors [who] called. Even a counselor call and another doctor were calling me over the phone. He said, "Okay, you're not suicidal." I said, "No, I'm not." Sometimes I'm just stressed out when I'm stressed out, sometimes, I just call one of the peach team and talk to them, and I feel much better.
		14	[...] the first person they sent was a psychiatrist [...] I see the psychiatrist every 2 weeks.
		14	Well, my mental health would not be as good [without PEACH] because the psychiatrist has been really, really great [...] I'd just be a lot more frantic and a lot more [...] It's good grounding to get a lot of stuff off my chest I can't say to other people.
		14	Interviewer: How were you taking care of your mental health before the PEACH psychiatrist?<br><br>Participant: I was afraid to touch it at all because I was afraid of getting locked up in a hospital again if I opened my mouth, so I was afraid to say anything to anybody.
Social Needs	*Connecting with Loved Ones*	3	PEACH ended up sending me and my family over to [a] restaurant. It was so nice, and they paid for the meal for all of us and the ride to get there and back. That was awesome. That was the last night before my family left, so it was a good ending to a three day stay.
		9	PEACH gave me hope. Additionally [...] my grandchildren are going to remember me you know. He's only 2 months old but he's going to remember me.

© 2026 Levesque AR et al. *JAMA Network Open*.

	Financial Support	1	They've figured out my [Ontario Disability Support Program] thing [...]
		1	[...] [The Health Navigator] is outstanding. Like going through I had real hard time with [the Ontario Disability Support Program] She just kept knocking at it till I got in. I got in like about a month ago.
		8	They're helping me get on disability because basically I can't do my job anymore and I never will be able to, and yea that's kind of really hard to take.
		8	They helped me get my [Ontario Disability Support Program] paperwork done [...]
		10	[...] They paid for my medications [...]
		12	[They help me with] my medication, sometimes when I can't afford it, and then they give me gift cards to purchase things. Food. Because they don't allow me to work, so they give me discounts [...]
		12	[...] the gift cards, they help me a lot when [they] send them to me, bring them to me, and I can go and shop [...] I know that I have something to eat, something to drink. I can give thanks and say the PEACH team is here for me because I'm not going to my bed hungry. I'm not going to say that that I'm hungry when they give me [a] gift card to go and shop.
		12	I will be down [without the PEACH team], because some of the time I can't buy my medication, as I have to buy my medication myself, and I'm not working. So sometimes, as I said, I get various aches and somebody might sponsor me. Or if I can't get dispensed, I'll call [the Health Navigator] and she would, she would make sure that I get it.
		14	They came and helped me fill out the disability tax credit, which was good [...] I asked every doctor to have it, and everyone said they were going to do it, and no one ever did it.
	Food Security	8	[...] the PEACH team got me [...] on a nutrition diet, I guess. Make sure that I'm putting enough calories in and stuff. So, it's good, they like make sure I get like boost and stuff delivered to the house.
		10	[The PEACH doctor], she was the one coming there, sending food, how do you call it, the chocolate stuff, and it was a bit cold that time, she brings me sleeping bags.
		12	[...] the gift cards, they help me a lot when [they] send them to me, bring them to me, and I can go and shop [...] I know that I have something to eat, something to drink. I can give thanks and say the PEACH team is here for me because I'm not going to my bed hungry. I'm not going to say that that I'm hungry when they give me [a] gift card to go and shop.
		12	I will be down [without the PEACH team], because some of the time I can't buy my medication, as I have to buy my medication myself, and I'm not working. So sometimes, as I said, I get various aches and somebody might sponsor me. Or if I can't get dispensed, I'll call [the Health Navigator] and she would, she would make sure that I get it.
		12	Interviewer: How would you get the food and drink you need without the peach team? Participant: Friends. Sometimes, sometimes I do eat out. Sometimes I will ask a friend, don't

© 2026 Levesque AR et al. *JAMA Network Open*.

			try to pressure no one for nothing. So, most important, I wait, and I wait until I get the gift card. If I don't get it for a while, I just ask a friend.
		2	Participant: I think it was through [the Nurse Coordinator] when I was at [the other shelter] and she was trying to get me into a bed because it was all cots there and you can't really sleep at all because once it's besides each other the whole place wakes up. So, she tried to get me in [this shelter] and she did. And after that she brought me to a couple of appointments for my cancer and that too and then she ended up helping me get a place.
		2	Just to get in this place and get a place, a lot of people wait years. I mean I've been in the shelter system for almost three years but still people [wait] sever, ten years.
		2	[The Nurse Coordinator] also took me to that to fill out the lease which was nineteen pages [...]
		2	We went in [the Nurse Coordinator's] car. I'm a neat freak, I'm very clean, but we went to see the apartment and it was amazing. Nice wooden floors and a good size bathroom. So tomorrow I'm going to check on the furniture.
		8	They're currently helping me right now with my house. Cause right now I moved with my brother and sister-in-law. Cause they live in a house and there's no stairs. They put me on a ground level of the house which is nice I have no stairs or anything like that and right now as we speak, I've actually gotten two applications in for two apartments. And yeah, that's through PEACH. PEACH has helped me out greatly on that. They got me through Toronto housing ahead of time and beautiful housing [...]
	Housing	9	PEACH found this place for me, this hospice, and I'm so happy. Like my apartment was dangerous for me, I'm a recovering addict, people are knocking on my door wanting to get high with me, it's really hard, I only have three months clean [...]
Relational Care	*Different Kind of People*	3	PEACH comes in as a palliative [team] to help you with what you need weekly or what you need right now. And that's not how [the cancer doctors] do it. They do it: just [come] here, take tests, won't tell you what's happening, and [then] here, try this.
		4	They come and see me too, the doctors actually come and see me, yeah, they reach out and they come and see me and that's amazing.
		4	And like you know, they don't bother you. They just help you, like they don't bother you saying, "you have to do this, and you have to do that, you have to hear me, and you have to do this." They don't do that you know. They just let you do what you got to do and [...] they cheer for you [...] That's what the saying is, they cheer for you.
		4	I really enjoy who they are individually who they are, I do I enjoy all of them, because they really put the positive in my life.
		4	I'm used to having like a doctor, I even had a surgeon way back when right, and I thought that was different, so this is like wow, seven doctors, it's like wow man, you really taking care of me.

© 2026 Levesque AR et al. *JAMA Network Open*.

		4	It's cool because I really do get along with them you know, and I do feel comfortable with every one of them and [...] I don't even think they try to be somebody else it's just who they are. Like you know they're just people, they just keep going, you know it's amazing [...] You know somebody makes a difference in your life, that's who they are. They made a difference in my life.
		4	Even when a I'm in a bad mood and pissed off, they're going to come and make me laugh.
		6	[...] nobody else is there for you when you need them no matter what, you know.
		7	[My PEACH doctor] is a good doctor [...] out of all the ones I dealt with, and there's some good ones, [he's] right up there on the top of the list.
		7	The best thing that I found about the PEACH team is the compassion that they had. I believe that everybody that I dealt with truly had the ambition to help. And that was the best thing, was that they wanted to help. And you don't find that everywhere, in hospitals, it's the choice of doctors and nurses. [The person] that does the hiring for the PEACH team hit the nail on the head. As I said, with compassion and care. Best thing. Knowing that somebody gives a shit, that's great. I said, "gives a shit."
		8	I've never really been a doctor person, because they're just always, I don't know, so sterile. And these people [from the PEACH team] are just like more caring and more welcoming.
		8	Yeah, they are amazingly caring. I'm not used to this, I've worked with 4000 other people and basically, you're told to f**k off all day long, sorry for the language but that's exactly what happens when I'm out in the world. But these people they come to my place, and I don't know they're just so, so good. And I'm not used to that. Anytime I've walked into a walk-in clinic or anything like that they're just so, so impersonal. And with these people it's just like they're caring!
		8	I consider [them] [...] you know a kind of friend. Even though they're my doctor, I can bond with [them] for sure.
		9	Like I said, I feel like they're my friends, I don't feel like they're working for me you, they're giving me a service. I think they're my friends.
		12	The peach team is a team that come in at home visits and they introduce themselves to me and started to talk. They tell me everything, encouraging me, talking to me, trying to tell me information about me, and then they encourage me about being active and not being sad. So, they always let me laugh and not feeling down or upset. Even if I'm upset, they come to see me. They would encourage me that they're there with me for a good while, talking to me, let me smile, and leaving me happy.
		12	[...] I feel love, I feel trust, and I can, I can say anything. I can ask them any question, and I can say anything, and they will give me an answer, and they're always there. So, the PEACH team is a blessing.

© 2026 Levesque AR et al. *JAMA Network Open*.

		12	They give you information. They test you; they tell you things. They just don't. For instance, the doctors just don't come and just tell you’re this and you're that and you are sick, no they talk to you. They communicate with you. You feel comfortable and feel like doing, listening, and answering their question that they asked, and then you can ask them questions [...] if you have anything to ask.
		13	Yeah, there’s three or four of them, they come by to say hi about once a month and see how I’m doing and [it] gives that family orientation thing you know, which is a good thing, I think.
		13	It was uh, it was kind of weird to me you know. I’m not used to that kind of introduction and friendliness, and I felt overwhelmed you know. It was a big sigh of relief, like finally I’ve got someone who cares, someone who wants to help, who hangs in there, who asks you, doesn’t go about it reluctantly. I haven’t [sensed] any reluctance in any of these people
		14	They [have] like a less stern outlook on life [...] they just [make] things easier you know [...] More approachable.
	Compassionate approach	2	Yeah, they’re definitely on my side, so that gives me a lot of support
		2	They’ve got my back maybe I should say.
		3	I have PEACH doing all the palliative care for me. Absolutely perfect, everything with them. They’re just, you know, gracious and really good with me, the nurses, the doctors.
		3	They came in and took charge of me and took care of me like I was you know, their only patient in a way. That’s how they made me feel. Like, it was like we’re going to make you feel better, we’re going to get you better and anything that came up he’d come in and he’d even show me things [...] and he'd say okay, I think I know what we could give you for that. No, there’s nothing I could change, everything they’ve done has been absolutely what I need.
		3	Between [PEACH] and [the housing staff], both of them. So good, so kind, both of them are so kind, it’s amazing. I’m not used to that kind of kindness inside [prison] because they’re always making things difficult for you.
		3	When they call its always so pleasant, if I call them, same thing. Call anytime they say, that makes you feel good knowing that they’re available all the time.
		4	They really kind, they’re generous, and they know what wrong with you before you do. It really is, true.
		4	Ahh geese, they’re there for you, if you need the help, they’re there to help you, if you need the help they’re there, and they, they’ll always be there too. They're great people.
		6	I know they’re there for me when I need them, anything medical or anything else, there’s always help somewhere.
		6	Just they’re willingness to help and do the best they can with what they have, which isn’t a whole lot.

© 2026 Levesque AR et al. *JAMA Network Open*.

		7	The best thing that I found about the PEACH team is the compassion that they had. I believe that everybody that I dealt with truly had the ambition to help. And that was the best thing, was that they wanted to help. And you don't find that everywhere, in hospitals, it's the choice of doctors and nurses. [The person] that does the hiring for the PEACH team hit the nail on the head. As I said, with compassion and care. Best thing. Knowing that somebody gives a shit, that's great. I said, "gives a shit."
		7	[...] like I said the compassion and care that the doctors and nurses had, and really, I believe wanted to help, do the best that's in their abilities to help me do the best I can to get through and have the best quality of life going towards the end of it. So that's the best thing.
		8	[...] it almost feels like I'm privileged you know what I mean? Like I don't know if I am, but it almost feels like I'm getting you know privileged, taken care of more. I hope everybody is getting taken care of, and I'm sure they are. It's just they're so [caring].
		8	I would love to keep the PEACH team. They're, they're something else, I tell you. They're really good.
		8	Yeah, they are amazingly caring.
		9	I'm going to tell you the thing that I've noticed the most is they really care about me; they really do, they really care about me. It's amazing. Especially the doctors and nurses.
		9	And this is the first time I can say I'm happy again. Since PEACH came into my life, I'm happy.
		9	That's what PEACH gave me. Hope. PEACH gave me hope.
		10	[The Health Navigator], she's strong, shown so much good leadership. She's understanding, she'll listen. Even when she's late or something, she's going to call you to either reschedule or do something. She'll get it done, those are some things I like about her, you know.
		12	Sometimes I'm just stressed out when I'm stressed out, sometimes, I just call one of the peach team and talk to them, and I feel much better.
		12	The PEACH team [...] I feel, I feel love, I feel trust, and I can, I can say anything. I can ask them any question, and I can say anything, and they will give me an answer, and they're always there. So, the PEACH team is a blessing.
		12	Sometimes I don't have no one to talk to [...] I could call [the PEACH team] and I say that hi how are you? I'm here and [they] make sure that I'm comfortable and smiling.
		12	It would be excellent for other people, at least they would have somebody around them to let them smile, somebody around and to let them feel comfortable, feel, even when they are hating themselves, they will see love again when PEACH team come around, they will feel love.
		13	They're more courteous, they're more compassionate, you know what I mean. Like compassionate frontline workers [...] They always try to make you feel good when they show up, and they do actually so people are happy to see them. I know I am.

© 2026 Levesque AR et al. *JAMA Network Open*.

	Wouldn't be here without PEACH	1	I wouldn't be here if it weren't for PEACH.
		1	Truthfully, I wouldn't be around if it wasn't for PEACH.
		1	I'd be dead. I would've given up. It's too complicated. Oh yea. For sure. There's not even a question in my mind.
		1	Oh, I would've given up. I would've given up after the wife passed. I was really in a bad place and having a cancer diagnosis is horrible. I would've given up for a 100% I would've given up for sure. No question about it I wouldn't made it through.
		2	I could be dead, who knows, huh, they've done so much for me and everything, it was like I had someone on my side.
		3	I wouldn't be where I am right now, not a chance. They're the ones that took the time to, nobody else thought about the power of getting my meds. The pharmacy didn't think about it. The doctors looked at it, but they didn't think about it. It was the doctor at PEACH who worked it out [...] I wouldn't be sitting up like this going and exercising, I'd still be laying on my bed sleeping most of the day and night, yeah.
		4	I think I'd be really hurting, for sure, I'd be really hurting. Yeah, cause they really, like I said I ain't got nothing bad to say like at all, they really, they made a big difference in myself [...] Cause I wouldn't know what to do with myself, like I said I'd just continue doing what I used to do, and it's like, it's just amazing, like I said I continued drinking really hard and I continue drinking really hard like I'm at three 26s a day and then some, you know what I mean, I was just trying to get out of my mind, just to get away, and all I do is really hurt myself like my livers gone, my whole lotta stuff in my stomach likes all screwed right up and as far as hope goes I got some. You know, I've got a lot of hope now.
		6	Interviewer: And if you were not served by the PEACH team, what do you think your health would be like right now?<br>Participant: Pretty lousy, I might not even be here right now.
		7	I don't know where I'd be without them. It got to the point where I could not function alone in my own apartment.
		8	They ended up hooking me up with PEACH and in all honestly thank God they did because PEACH has helped me greatly so far. I can't imagine where I'd be this far ahead without PEACH.
		9	I wouldn't be here today if it wasn't for PEACH. I'd be dead.
		9	I don't think I'd be here, I really don't.
		9	I'm not sure what I'd have done you know. I was ready to do [Medical Assistance in Dying].
		10	Regarding my presence here, it's because of PEACH I'm here.

© 2026 Levesque AR et al. *JAMA Network Open*.

		13	[...] if it wasn't for them, I wouldn't be nowhere near where I am.
		13	Like Jesus, I'd be nowhere near where I am, I could be dead right now.

© 2026 Levesque AR et al. *JAMA Network Open*.

eTable 3. Supplementary Quotes – Constructive Feedback

Sub-Theme	Participant Number	Quote
Gratitude	1	They dealt with it right away. Everything. Everyone I dealt with peach so far have been outstanding in their job.
	1	Interviewer: Are there any areas that you feel like that they could improve or anything that you think that they could work on as a team together?<br>Participant: No, I really don't, I really don't. Every aspect so far has been outstanding like really, I'm not just saying that.
	2	You're [the PEACH team] fantastic. It's too bad, I wish every person that has cancer had this palliative care.
	2	I'm glad I had a chance to talk about PEACH, how great they are. I'm really, really happy with them.
	4	Yeah, they're helping me with everything, yes, they're helping me with everything. Name it, they help me with it. They're just unbelievable, to me they are, to me they're just great.
	4	They're just great, I've got not a bad word to say about them, not a bad word to say about any one of them, they're all great.
	4	The only thing is, I don't really know how I got the connection, but I did, I really did. And that was beautiful to. You know I don't even use these words normally, beautiful [...] t's just I don't even know how we got connected but I'm happy I did, because it's all been positive, it's all been good.
	4	I love them, they're great. That's it.
	6	Yeah, so they help me out a lot. Actually [the Nurse Coordinator] was unbelievable. She was just… and [the new coordinator] seems like [...] she's fitting in really well. So yeah, other than that, I mean the doctor I had when I first went on was, I don't know if you know him or not [...] Oh my god, he was amazing, and unfortunately, it wasn't long after he started working with me, he took off and went to do his thing [...]
	7	[...] there is nothing that they could've done. They made no errors in any of the decisions they made as far as my care goes. And we will keep it at that. They made no mistakes. I do follow-up, I do research, and yeah there's nothing that they could've done differently than what they did, if there is I didn't find it.
	8	And you know I've never been a doctor person and they're making it easy for now which I appreciate.
	8	If they need more funding, I think they should get it. You know I won the lottery with them, and I think they should deserve 1 million times more [funding]. I think they're doing great work.
	9	I've been with PEACH ever since the doctor told me I was palliative and I'm so grateful. They give me 18 months to live, I bypassed that long ago.
	9	I'm so grateful to PEACH, when I tell you I'm happy. You can tell. When I talk about PEACH, I'm like [...] I love them. I love them.
	10	I think the PEACH team has been a blessing to me… but you're good, you're good. It's well established, well established.

© 2026 Levesque AR et al. *JAMA Network Open*.

	10	Very good establishment. Keep it up.
	12	I see the team as a blessing in my life.
	12	I can give thanks and say the PEACH team is here for me because I'm not going to my bed hungry.
	12	[...] they're doing a wonderful job [...] I wouldn't change anything. Just give them [my] blessing and [I'm] grateful for them, thankful for them.
	12	The only thing I have to say is [I'm] sending a prayer for you guys in the PEACH team and continue to do what you're doing, because you guys are doing a wonderful job. And I thank God for the day when I met you guys, I'm grateful for it, because you open up doors for me and help me a lot. So, I'm grateful and thankful, and I don't want nothing to change about it, [except it] must be upgraded more to every other person.
	13	I'm very thankful that they're even there. It's overwhelming to come from the old school into this bright new thing you know, but yeah it just made me so happy to know not even so much that they're going to be doing something for me just that people care you know [...] and they come in all smiles ready to be my front line through the war. I thought that was awesome.
	13	Interviewer: If you had a magic wand and could do anything with regards to the PEACH team, what would you do?<br>Participant: Buy them lunch every day.
	13	Just a really super huge hug and thank you. I mean that too, I really do mean that. I haven't been so serious in my life until this point and just that, you know, for the extra couple of years I may get.
	14	I really appreciate them, and I wish them the best of luck [...] I hope that things just get better and better.
PEACH Would Be a Good Service for Others	1	[PEACH would be good for] even the people that are housed that don't have like a very good support system. Sure, you should expand it.
	1	If there's a guy that's not sure [about PEACH], get him to call me and I'll switch him around you guys were GREAT!
	2	I've told other guys who've complained about what was happening with them and you know what you should phone this number and see if you can get in with the PEACH team and tell them about your problems. I've said that to a few people here [...]
	6	I wish there were a whole pile of people who were given that service. You know I go to hospital, and I say I'm with the PEACH program, "Who's that?", nobody seems to know, so you know I try to explain it to them, educate them. But you can't go ahead and start referring people, you know, it's just not the way it's done, so I hope it is done in another way, I hope they do find another way for getting, I know they're probably in a really tough spot right now you know because there's so many people other there who need the help and there's only so many of them and they're all volunteers so I'm sure it's pretty hard sometimes to get everything together and get help for everybody, that's sort of why I'm glad I'm in at, you know, the sort of ground level type thing. Yeah, so, because I don't know if people can still get, that type of help.

© 2026 Levesque AR et al. *JAMA Network Open*.

	9	Interviewer: Any other kinds of people you feel PEACH would be good for?<br>Participant: Well homeless people, homeless people. People who are struggling to come off of a drug. Especially for women. Men have many, many resources out there. Not women, not women.
	14	I have one friend who was just in hospital, and she had pneumonia [...] and I just think why can't these great people who sort of come to the building and I could just point her out and say “she needs to be talked to” I think they could've made a huge difference, and she wouldn’t be dead right now.
Areas for Improvement	6	My memory is really bad so that’s one thing they are not helping me with, and I keep telling them I’m having a problem.
	6	I wish there were a whole pile of people who were given that service. You know I go to hospital, and I say I’m with the PEACH program, “Who’s that?”, nobody seems to know, so you know I try to explain it to them, educate them…
	7	It is the only downside […] that if I had the money or funds to improve it, I would improve that part of the program to have somebody available to give [in person] care after hours [...] Like a nurse or a nurse practitioner who could come over. Sometimes it’s just a comfort thing. The things I suffer from, the exacerbations of my breathing mostly happen at night […] Where having somebody around that could calm me down, get me through the panic attack as well as the exacerbation, because they kind of go hand-in-hand and it’s terrible to go through both of those things, and to have somebody there [in-person] to help through that would be really comforting.
	7	I mean to have a regular doctor look after your needs rather than different ones all of the time would be great, but I realize that funding is problem, there’s not enough doctors to go around, nurses to go around, and I understand all of that, so for someone like myself who can comprehend all that, you’ve got to accept that.
	9	The only thing I find hard is that every time I have different doctors, and you feel like you have to tell your story again. You get kind of tired [of] telling your story.
	13	I don’t think anyone around here has heard of the PEACH team I almost think they’re brand new.

© 2026 Levesque AR et al. *JAMA Network Open*.
