# Supplementary material for: Patient Perspectives on Palliative Care Outreach to Adults Living With Homelessness
Source: JAMA Netw Open. 2026 Jan 15;9(1):e2552434. doi: 10.1001/jamanetworkopen.2025.52434 (PMC12809365; doi:10.1001/jamanetworkopen.2025.52434)
Supplement: Supplement 2. — Data Sharing Statement [file jamanetwopen-e2552434-s002.pdf]

Data Sharing Statement

Levesque. Patient Perspectives on Palliative Care Outreach to Adults Living With Homelessness. *JAMA Netw Open*. Published January 15, 2026.
doi:10.1001/jamanetworkopen.2025.52434

Data
Data available: No

Additional Information
Explanation for why data not available: Patient demographics are available in the body of the manuscript. Extensive supplementary quotes are included in the Online Only Material. Complete transcripts are not available due to concerns this could lead to patient identification.
